# From Subtle Signs to Severe Sequelae—A Century of Symptomatology and Comorbidities in the Diagnosis of GH-Secreting Pituitary Neuroendocrine Tumors: A Systematic Review

**DOI:** 10.3390/diagnostics15172137

**Published:** 2025-08-24

**Authors:** María José Ayora, Lizeth Vinueza-Mera, Santiago Aynaguano, David Poma Jimenez, Felipe Loza Hernandez, Sebastian Jara Jimenez, Jose A. Rodas, Jose E. Leon-Rojas

**Affiliations:** 1 NeurALL Research Group, Quito 170157, Ecuador; mjayora98@gmail.com (M.J.A.); santiago.aynaguano@unach.edu.ec (S.A.); dr.davidpoma@gmail.com (D.P.J.); felipaoloza@gmail.com (F.L.H.); sebastianjara_2000@outlook.com (S.J.J.); 2 Escuela de Medicina, Universidad de las Américas (UDLA), Quito 170124, Ecuador; lizeth.vinueza.mera@udla.edu.ec; 3 Escuela de Medicina, Universidad Nacional de Chimborazo, Riobamba 170518, Ecuador; 4 Escuela de Medicina, Universidad Nacional de Loja, Loja 110103, Ecuador; 5 Escuela de Medicina, Universidad Internacional del Ecuador, Quito 170411, Ecuador; 6 School of Psychology, University College Dublin, D04 V1W8 Dublin, Ireland; josea.rodasp@gmail.com; 7 Escuela de Psicología, Universidad Espíritu Santo, Samborondón 092301, Ecuador

**Keywords:** gigantism, acromegaly, signs, symptoms, comorbidities, growth hormone-secreting PitNET, pituitary neuroendocrine tumors

## Abstract

**Background/Objectives**: Somatotropinomas rank as the second most prevalent functional pituitary neuroendocrine tumors (PitNETs), responsible for acromegaly in adults and gigantism in children. Early diagnosis and treatment would help prevent irreversible physical changes and other associated comorbidities. The aim of this review is to characterize the symptomatic presentation of growth hormone (GH)-secreting PitNET at the time of diagnosis. **Methods**: A search was conducted in PubMed, Scopus, Cochrane, and the Virtual Health Library (VHL). Primary descriptive and analytical studies were selected if they were written in Spanish or English and addressed the symptoms of acromegaly and/or gigantism due to somatotropinomas. **Results**: Out of 8470 articles, 93 fulfilled the inclusion criteria, covering 1745 patients (55.4% women). The most frequent diagnostic signs/symptoms were enlarged extremities (12.4%) and facial changes (13.1%). Endocrine–metabolic (42.82%) and cardiovascular (31.45%) were the most prevalent comorbidities. The average diagnostic delay was 6.7 years, with the number of reports of the disease significantly increasing in recent decades, most likely due to ongoing advances in imaging and standardized hormonal tests. **Conclusions**: Timely recognition of a somatotropinoma’s symptoms and comorbidities is crucial for early diagnosis and referral to specialized care and the prevention of permanent physical and/or physiological changes.

## 1. Introduction

Gigantism has been described in legendary tales, depicted in art throughout history, and found in fossil records, suggesting its presence for a long time in different populations [1]. However, gigantism was not studied as a pathological entity until 1864 when Andrea Verga described a “destroyed sphenoid bone and displaced optic chiasm by a walnut-sized sellar tumor” in a postmortem examination of a woman with dysmorphic facial features [1]. In 1886, Pierre Marie coined the term acromegaly to describe the disease, and its etiology was attributed to a pituitary enlargement by Minkowski in 1887 [2]. Gigantism and acromegaly were, generally, not considered the same disease until 1895, with gradual acceptance increasing as pathophysiology was better understood [2,3]. It was with the advancement of the fields of microscopy, imaging, and endocrinology that in 1900 Benda determined the association of gigantism/acromegaly with the pituitary eosinophilic tumor and hyperfunction of the gland [3]. As the field of molecular biology developed in 1921, Evans and Long demonstrated the production of growth hormone (GH) in the pituitary gland and were able to show similar changes in animal studies; later, in 1950, Li and Papkoff were able to isolate GH [1].

Pituitary neuroendocrine tumors (PitNETs), previously known as pituitary adenomas, are the most frequent benign tumors of the sellar region and are classified as microadenomas (micro-PitNETs) when the measure is less than 10 mm in diameter, or macroadenomas (macro-PitNETs), when the measure is greater than 10 mm in diameter [4]; of these, GH-secreting PitNETs account for approximately 20% and are benign neoplasms that lead to excess secretion of the hormone, manifesting as two clinical syndromes: acromegaly and gigantism [5]. Acromegaly manifests in adults once the epiphyseal plates have closed, preventing the longitudinal growth of long bones and leading to a disproportionate increase in soft tissues and bones of the extremities [6]. This disorder not only affects physical appearance, but is also associated with multiple metabolic and systemic complications or comorbidities, such as diabetes mellitus, obstructive sleep apnea, hypertension, and cardiovascular diseases [7]. Additionally, patients with acromegaly have a higher risk of developing malignant neoplasms [7]. In contrast, gigantism occurs in children and adolescents before the closure of the epiphyseal plates, allowing for exaggerated height growth. Children and adolescents affected by gigantism experience rapid and excessive growth in stature, as well as physiological alterations similar to those observed in advanced stages of acromegaly; this uncontrolled growth can lead to musculoskeletal problems, respiratory difficulties, and other health complications [8].

The primary objective of this study is to examine the clinical signs and symptoms, as well as the comorbidities, present at the time of diagnosis of GH-secreting PitNETs in the literature. With this, we aim to incentivize discussion in the medical and research community to inspire future studies to improve early detection of these tumors. To achieve this, we will evaluate the frequency and chronology of these manifestations and identify clinical patterns that may serve as early indicators of the disease. Additionally, we will organize the collected data by geographical region to analyze how socioeconomic factors and access to healthcare, typical of each region, might influence the timing of diagnosis and the severity of clinical presentation.

## 2. Materials and Methods

This systematic review was registered in PROSPERO (CRD42022378791) and followed the 2020 Preferred Reporting Items for Systematic reviews and Meta-Analyses (PRISMA) guidelines (Table S1).

### 2.1. Eligibility Criteria

We compiled data from PubMed (MEDLINE), Scopus, Cochrane Central Register of Controlled Trials (CENTRAL), and the Virtual Health Library (VHL), from inception until 13 November 2022. Inclusion criteria included original observational studies (cross-sectional, cohort, case–control, case series, or case reports), in Spanish or English, with participants with a confirmed and complete diagnosis somatotropinoma (i.e., with imaging confirmation of a PitNET, laboratory confirmation of elevated GH, and description of symptomatology/complications at the time of the diagnosis). Studies were excluded if the following occurred: (1) patients received any form of treatment; (2) other pituitary hormones were affected; (3) the studies were letters to the editor, congress abstracts, the literature, scoping or systematic reviews, animal-based models, or in vitro studies; and (4) the patients were diagnosed with other endocrine syndromes that could cause somatotropinomas.

### 2.2. Search Strategy

The following medical subheadings (MeSH) and search terms were used: hyperpituitarism, acromegaly, gigantism, growth hormone, signs, and symptoms; therapy and treatment were excluded. A detailed description of the search terms used and the specific search strategy used in each database can be found in the Supplementary Materials (Section S1).

### 2.3. Data Management

The resulting articles from the databases were handled using the web-based software Rayyan, developed by the Qatar Computing Research Institute (https://www.rayyan.ai/, accessed on 15 December 2022). Rayyan mitigates data entry errors, avoids mistakes during the removal of duplicate articles, and reduces the risk of bias during the selection and decision process. Duplicates were detected and removed by using this software; confirmation of the duplicates and elimination was performed manually by the reviewers.

### 2.4. Selection Process

All abstracts, titles, and keywords were analyzed by two double-blinded teams that applied the aforementioned eligibility criteria. Discrepancies were solved by two members of the team not previously involved in this process, and by discussion and mutual consensus of the reviewers. Articles that surpassed this first filter were then subjected to a full-text analysis (against eligibility criteria) for the final selection of the articles to be included in the review.

### 2.5. Data Items and Data Synthesis

The articles that passed all filters were then subjected to data extraction; the following variables were recollected from each article: age at diagnosis, age at symptomatic onset, gender, insulin-like growth factor 1 (IGF-1) value and index, GH value, tumor size, country of publication, year of publication, symptoms, and comorbidities. Years of diagnostic delay were calculated as the difference between the age at diagnosis and the age at symptom onset. For the analysis of the data collected, we separated the patients based on the age of symptom onset to discriminate between patients with acromegaly and gigantism; we determined the cut-off point at 18 years of age. However, it is important to note that there is no exact cut-off age for epiphyseal fusion and growth senescence reported in the literature; it typically occurs in late adolescence, after the progressive decline in growth rate, but is also influenced by the individual’s hormonal changes (mainly estrogen, growth hormone, and IGF-1) [9].

Symptoms and comorbidities were grouped based on a comprehensive list of body systems. This categorization was employed to comprehensively reflect the multisystemic nature of GH-secreting PitNETs and their varied clinical manifestations, including physical changes and numerous systemic complications that affect different organ systems in both acromegaly and gigantism. The percentages of these were obtained based on the relative frequency of reporting in the articles. Relevant laboratory results were also extracted, and all data were converted to the same unit of measurement to enable comparisons. The articles were grouped by decade based on the year of publication, and null data was omitted to ensure a clearer visualization and more accurate representation in the graphical analysis. Weighted averages were used for each decade’s bracket, and standard deviations were obtained. Studies were also classified by region based on the World Health Organization’s distribution.

### 2.6. Risk of Bias Assessment

We assessed risk of bias in the included studies using the Study Quality Assessment Tools from The National Heart, Lung, and Blood Institute (NHLBI) (https://www.nhlbi.nih.gov/health-topics/study-quality-assessment-tools, accessed on 26 November 2023). We identified the type of study, selected the appropriate tool, and then answered each tool’s questions with Yes, No, or Unclear. For case reports, we used the Quality Assessment Tool for Case Series Studies but eliminated questions 3, 4, and 8, as they do not apply to individual case reports.

Two reviewers independently assessed every article, and each bias assessment was recorded on an information storage platform to enable comparisons. Discrepancies in the level of bias were resolved by a third reviewer acting as an arbiter and after mutual consensus. Following the NHLBI guide, we derived an overall summary judgment (low, moderate, or high risk of bias) for each study. The overall risk of bias for each study was determined by the highest risk level identified in any of the domains assessed. A study was considered as having a low risk of bias if it received a positive answer to 80% or more of the questions; if it received a positive answer in 50–79% of the questions, it was classified as moderate risk of bias; and if it received a positive answer to less than 50% of the questions, it was classified as a high risk of bias.

## 3. Results

### 3.1. Study Selection

**Figure 1:**
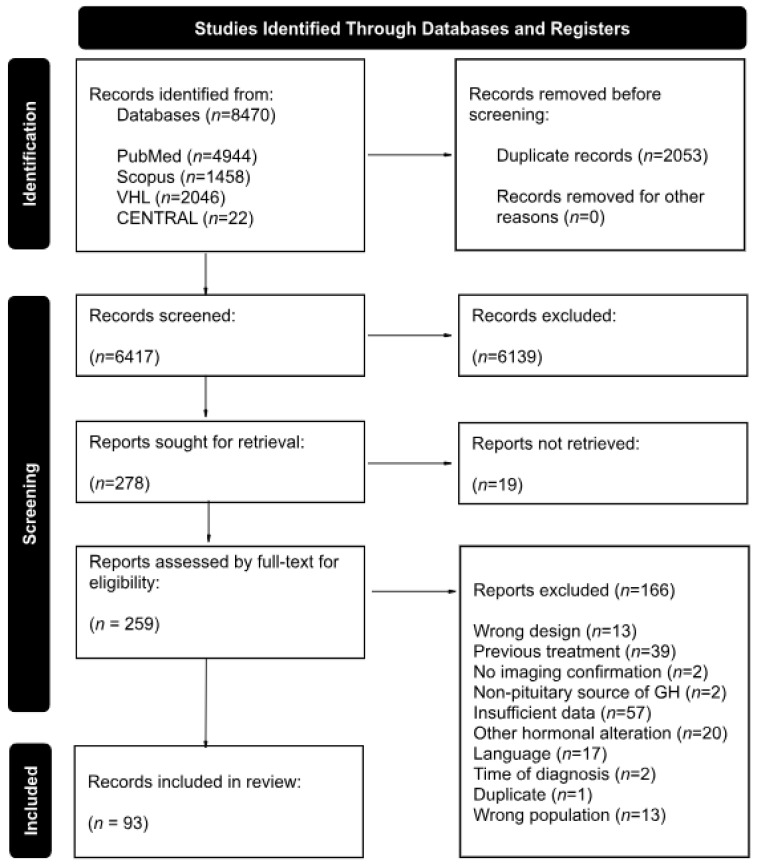
Flowchart of study selection according to the inclusion and exclusion criteria, in strict adherence to the PRISMA 2020 guidelines.

The literature search yielded a total of 8470 records from the four databases; after duplicate removal and analysis of the abstract, title, and keywords, a total of 259 records were included for full-text analysis, from which 93 articles were ultimately included in our review representing a total of 1745 participants (55.4% female) [10,11,12,13,14,15,16,17,18,19,20,21,22,23,24,25,26,27,28,29,30,31,32,33,34,35,36,37,38,39,40,41,42,43,44,45,46,47,48,49,50,51,52,53,54,55,56,57,58,59,60,61,62,63,64,65,66,67,68,69,70,71,72,73,74,75,76,77,78,79,80,81,82,83,84,85,86,87,88,89,90,91,92,93,94,95,96,97,98,99,100,101,102]. Figure 1 showcases the complete selection process.

The studies encompassed multiple geographical regions, with the United States (22 studies) and England (11 studies) being the primary contributors; Japan (8 studies), Italy (7 studies), Türkiye (7 studies), and India (7 studies) also had notable representation. Additional studies were conducted in various other countries; a detailed breakdown by study type can be found in Table 1.

### 3.2. Risk of Bias Findings

Regarding study design, case series were the most prevalent, accounting for 55 studies of our included studies, followed by cross-sectional studies (*n* = 20 articles), case–control studies (*n* = 11 articles), and case reports (*n* = 7 articles). When looking at the risk of bias, 64 studies had a low risk of bias, 27 were rated as moderate risk of bias, and only 2 articles were considered to have a high risk of bias (both case reports with incomplete reporting of relevant information). Although most studies exhibited a low risk of bias, those classified as moderate risk often had limitations in confounder assessment, outcome measurement, and handling of missing data. Table 1 has a complete breakdown of the bias assessment as well as relevant characteristics of all the included articles.

### 3.3. Temporal and Geographical Analysis

**Figure 2:**
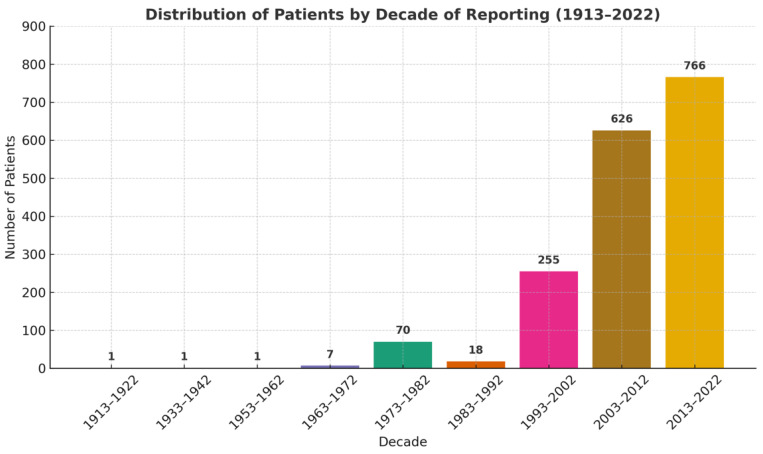
Number of patients reported by decade from 1913 to 2022.

In this review, we wanted to provide insight into the number of reported patients in the literature over time. The collected articles comprehend data from 1913 to 2022 [10,11,12,13,14,15,16,17,18,19,20,21,22,23,24,25,26,27,28,29,30,31,32,33,34,35,36,37,38,39,40,41,42,43,44,45,46,47,48,49,50,51,52,53,54,55,56,57,58,59,60,61,62,63,64,65,66,67,68,69,70,71,72,73,74,75,76,77,78,79,80,81,82,83,84,85,86,87,88,89,90,91,92,93,94,95,96,97,98,99,100,101,102]. In the first 50 years (1913–1962), only one patient was reported per decade (Figure 2). Afterwards, reporting steadily increased since the 1990s with 255 cases. In the 2000s, the reported cases more than doubled (626 cases), and in the most recent decade, the number tripled (766 cases) compared to 1993–2002 [10,11,12,13,14,15,16,17,18,19,20,21,22,23,24,25,26,27,28,29,30,31,32,33,34,35,36,37,38,39,40,41,42,43,44,45,46,47,48,49,50,51,52,53,54,55,56,57,58,59,60,61,62,63,64,65,66,67,68,69,70,71,72,73,74,75,76,77,78,79,80,81,82,83,84,85,86,87,88,89,90,91,92,93,94,95,96,97,98,99,100,101,102].

**Figure 3:**
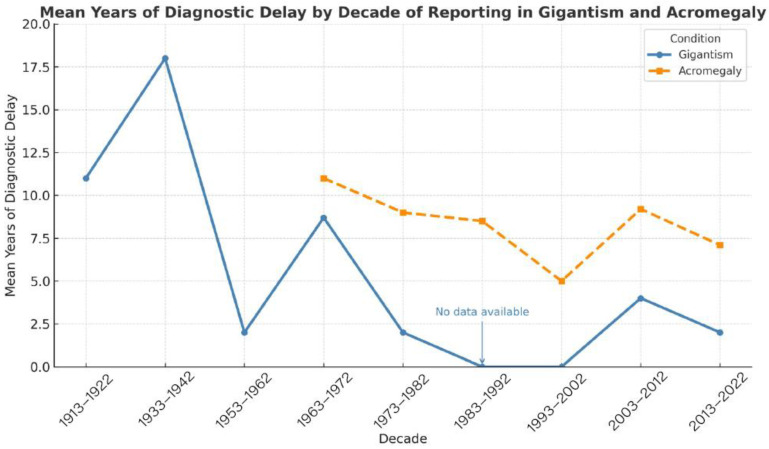
Mean years of diagnostic delay in patients with gigantism and acromegaly compared, by decade of literature reporting.

A total of 1745 patients were included in our study, of which only 11 were diagnosed with gigantism and the rest with acromegaly [10,11,12,13,14,15,16,17,18,19,20,21,22,23,24,25,26,27,28,29,30,31,32,33,34,35,36,37,38,39,40,41,42,43,44,45,46,47,48,49,50,51,52,53,54,55,56,57,58,59,60,61,62,63,64,65,66,67,68,69,70,71,72,73,74,75,76,77,78,79,80,81,82,83,84,85,86,87,88,89,90,91,92,93,94,95,96,97,98,99,100,101,102]. We wanted to also analyze the temporal trends of reporting in the scientific literature with regard to the diagnostic delay of gigantism and acromegaly (i.e., the mean year difference between the actual onset of the disease and the medical diagnosis, as reported in the literature). Figure 3 showcases the mean years of diagnostic delay in both gigantism and acromegaly patients stratified by decades of scientific literature reporting [10,11,12,13,14,15,16,17,18,19,20,21,22,23,24,25,26,27,28,29,30,31,32,33,34,35,36,37,38,39,40,41,42,43,44,45,46,47,48,49,50,51,52,53,54,55,56,57,58,59,60,61,62,63,64,65,66,67,68,69,70,71,72,73,74,75,76,77,78,79,80,81,82,83,84,85,86,87,88,89,90,91,92,93,94,95,96,97,98,99,100,101,102]. It is important to note that, as technology has advanced, the diagnostic delay has decreased somewhat steadily over the years; however, it is striking that it has not reached zero in the published literature.

When looking at the age of onset of the disease, in most cases of gigantism the onset of symptoms is at the pre-teen and teen years; however, none of our included papers presented a case of growth hormone-secreting PitNET during infancy and early childhood [10,11,12,13,14,15,16,17,18,19,20,21,22,23,24,25,26,27,28,29,30,31,32,33,34,35,36,37,38,39,40,41,42,43,44,45,46,47,48,49,50,51,52,53,54,55,56,57,58,59,60,61,62,63,64,65,66,67,68,69,70,71,72,73,74,75,76,77,78,79,80,81,82,83,84,85,86,87,88,89,90,91,92,93,94,95,96,97,98,99,100,101,102]. The latest reported onset of gigantism in our data set was at 16 years old, and the youngest 7 years old. In contrast, acromegaly patients tend to be middle-aged adults; the oldest mean age of onset in our data set was 44.2 years old and the youngest 32.6 years old [10,11,12,13,14,15,16,17,18,19,20,21,22,23,24,25,26,27,28,29,30,31,32,33,34,35,36,37,38,39,40,41,42,43,44,45,46,47,48,49,50,51,52,53,54,55,56,57,58,59,60,61,62,63,64,65,66,67,68,69,70,71,72,73,74,75,76,77,78,79,80,81,82,83,84,85,86,87,88,89,90,91,92,93,94,95,96,97,98,99,100,101,102].

**Figure 4:**
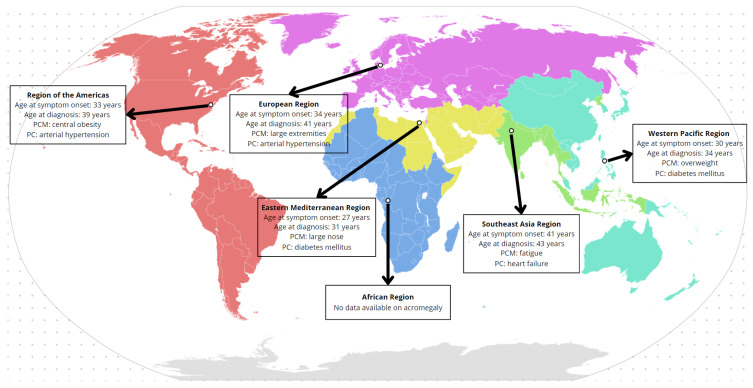
Predominant clinical manifestation (PCM) and predominant comorbidity (PC) of acromegaly organized by WHO regions.

Finally, relevant demographic, biochemical, and clinical variables were collected from all articles; a comprehensive table with all these characteristics is provided in the Supplementary Materials (Table S2). After analyzing the literature’s temporal trends, we conducted a geographical analysis to determine the predominant clinical manifestation (PCM) and predominant comorbidity (PC) of those diagnosed with acromegaly and stratified according to the World Health Organization (WHO) regions as shown in Figure 4.

Out of the six WHO regions, data from five were included in our analysis (as we only found one article in the African region from 1968, looking at gigantism only [81]). To begin with, the age of onset of symptoms varies importantly by region. Patients from the Eastern Mediterranean region are the first to report clinical manifestations, with an average age of onset of 27 years [27,38,71,80]. In contrast, patients from the Southeast Asia region presented symptoms later, with an average age of onset of 41 years [28,30,32,45,55,79,89,92,93,96].

The most frequently observed PCM was abnormal growth in certain regions of the body, including enlarged extremities and a prominent nose, with more frequency in the Europe and Eastern Mediterranean regions; other relevant PCMs included overweight, central obesity, and fatigue [10,11,12,13,14,15,16,17,18,19,20,21,22,23,24,25,26,27,28,29,30,31,32,33,34,35,36,37,38,39,40,41,42,43,44,45,46,47,48,49,50,51,52,53,54,55,56,57,58,59,60,61,62,63,64,65,66,67,68,69,70,71,72,73,74,75,76,77,78,79,80,81,82,83,84,85,86,87,88,89,90,91,92,93,94,95,96,97,98,99,100,101,102]. Notably, the most common PCs were diabetes mellitus and hypertension, each dominating in distinct regions; in the Southeast Asian region, heart failure stands out as the leading comorbidity [10,11,12,13,14,15,16,17,18,19,20,21,22,23,24,25,26,27,28,29,30,31,32,33,34,35,36,37,38,39,40,41,42,43,44,45,46,47,48,49,50,51,52,53,54,55,56,57,58,59,60,61,62,63,64,65,66,67,68,69,70,71,72,73,74,75,76,77,78,79,80,81,82,83,84,85,86,87,88,89,90,91,92,93,94,95,96,97,98,99,100,101,102]. Finally, regarding diagnostic delay, patients from the Southeast Asia region experience a delay of 2 years, while in the European region, the delay was greater, at 7 years [10,11,12,13,14,15,16,17,18,19,20,21,22,23,24,25,26,27,28,29,30,31,32,33,34,35,36,37,38,39,40,41,42,43,44,45,46,47,48,49,50,51,52,53,54,55,56,57,58,59,60,61,62,63,64,65,66,67,68,69,70,71,72,73,74,75,76,77,78,79,80,81,82,83,84,85,86,87,88,89,90,91,92,93,94,95,96,97,98,99,100,101,102].

### 3.4. Frequency of Signs, Symptoms, and Comorbidities at Diagnosis

**Figure 5:**
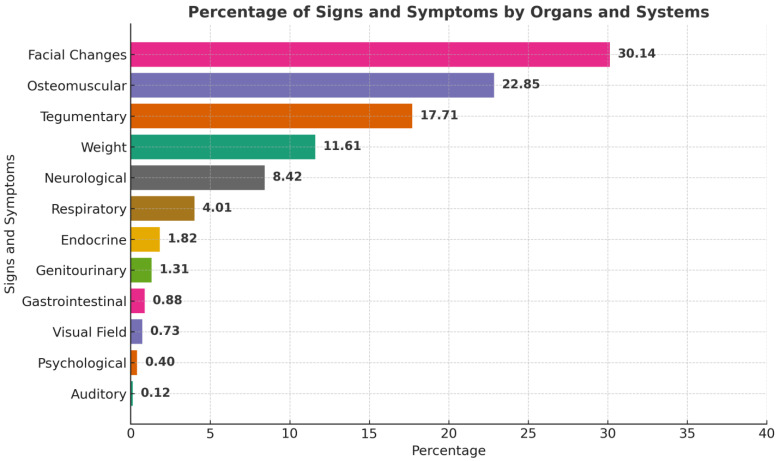
Percentage of signs and symptoms reported by organ and system at the time of diagnosis.

The most common signs and symptoms reported in the literature were facial changes and those associated with the osteomuscular and tegumentary systems; the least frequent were visual, psychological, and auditory signs and symptoms (Figure 5) [10,11,12,13,14,15,16,17,18,19,20,21,22,23,24,25,26,27,28,29,30,31,32,33,34,35,36,37,38,39,40,41,42,43,44,45,46,47,48,49,50,51,52,53,54,55,56,57,58,59,60,61,62,63,64,65,66,67,68,69,70,71,72,73,74,75,76,77,78,79,80,81,82,83,84,85,86,87,88,89,90,91,92,93,94,95,96,97,98,99,100,101,102]. Table 2 and Table 3 showcase the absolute frequency of relevant signs and symptoms reported at diagnosis of acromegaly and gigantism, respectively; Figure 5 provides a graphical representation of the relative frequencies of these signs and symptoms but grouped by relevant bodily systems. If the reader wants more information regarding specific manifestations at diagnosis, please review the Supplementary Materials where we present a more granular and specific list (Table S3).

**Figure 6:**
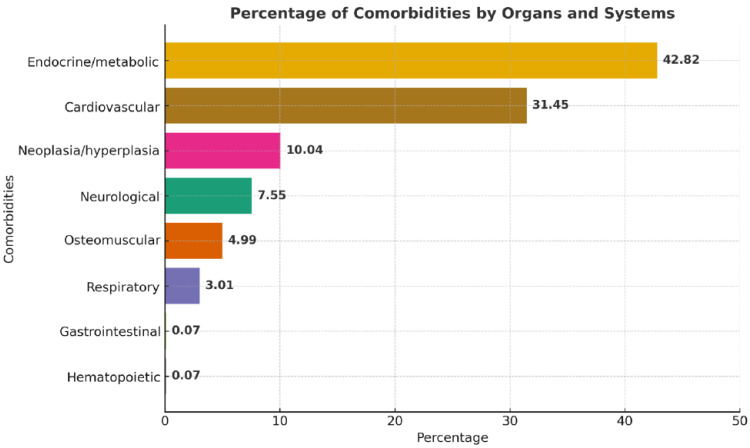
Percentage of comorbidities reported by organ and system at the time of diagnosis.

On the other hand, when looking at the reported comorbidities at diagnosis, the most commonly presented were endocrine–metabolic, cardiovascular, and neoplasia/hyperplasia; the least frequent were visual, respiratory, gastrointestinal, and hematopoietic comorbidities [10,11,12,13,14,15,16,17,18,19,20,21,22,23,24,25,26,27,28,29,30,31,32,33,34,35,36,37,38,39,40,41,42,43,44,45,46,47,48,49,50,51,52,53,54,55,56,57,58,59,60,61,62,63,64,65,66,67,68,69,70,71,72,73,74,75,76,77,78,79,80,81,82,83,84,85,86,87,88,89,90,91,92,93,94,95,96,97,98,99,100,101,102] (Figure 6). Table 4 and Table 5 showcase the absolute frequency of comorbidities of acromegaly and gigantism, respectively; Figure 6 provides a graphical representation of the relative frequencies of these comorbidities but grouped by relevant bodily systems. If the reader wants more information regarding specific comorbidities, please review the Supplementary Materials where we present a more granular and specific list (Table S4).

### 3.5. Diagnostic Results

Finally, when looking at the reported diagnostic tests performed on the reported participants, not all studies reported hormonal or imaging results, and on the ones that did, each result was reported based on the laboratory’s normal range and preferred units [10,11,12,13,14,15,16,17,18,19,20,21,22,23,24,25,26,27,28,29,30,31,32,33,34,35,36,37,38,39,40,41,42,43,44,45,46,47,48,49,50,51,52,53,54,55,56,57,58,59,60,61,62,63,64,65,66,67,68,69,70,71,72,73,74,75,76,77,78,79,80,81,82,83,84,85,86,87,88,89,90,91,92,93,94,95,96,97,98,99,100,101,102]; these were all unified and converted to the same unit. Table 6 showcases the diagnostic results reported in the literature stratified by decade of publication.

When analyzing only for the subgroup of patients with gigantism only three publications reported IGF1 with a mean of 1328.3 (μg/L) and IGF1 index of 3.0, seven publications reported GH with a mean of 17.8 (μg/L), and three publications reported tumor size with a mean of 8 mm, range 4–12 mm.

## 4. Discussion

The increase in reported cases of GH-secreting PitNETs in our temporal analysis could be the result of advancements in diagnostic techniques and better access to medical services. Certainly, the higher numbers in the last two decades, according to our results, may reflect a rise in the incidence due to a wider use of imagining techniques and improved reporting practices for these patients [103]. The rise in reported cases might also correlate with an increase in symptom documentation. Studies looking at the prevalence of symptoms, without regard to time of diagnosis nor time since symptom onset, report a prevalence of mandibular growth of 22 to 24%, prognathism of 20 to 22%, macroglossia of 54 to 58%, and diastema of 40 to 43% [4,5,6,7,8]. A systematic review published in 2023 reported the most common symptom at diagnosis was acral enlargement with a prevalence of 90%, followed by alteration of facial features with 65%, and oral changes with 62% [8]. Our systematic review found a prevalence of change in facial features of 30.13% that included mandibular changes, diastema, large lips, large nose, macroglossia, and prominent features, followed by osteomuscular changes in 22.83% that included abnormal height, arthralgia, weakness, bone hypertrophy, and extremities’ soft tissue hypertrophy. An important difference lies in our rigorous inclusion criteria: not including studies involving mixed GH/PRL-secreting PitNETs or other hormonal syndromes to focus exclusively on GH-secreting somatotropinomas. We acknowledge that mixed PitNETs often present with similar symptomatology and comorbidity burden, but we opted for a narrower scope to reduce hormonal confounding and work with a more homogenous population. Furthermore, we found that the distribution of signs and symptoms across the five WHO regions is heterogeneous, with certain clinical manifestations being more prevalent in some regions than in others. These factors directly influence both the number of diagnosed patients and the delay in diagnosis. For example, in Ecuador, the reported prevalence is 18 cases per million inhabitants, a significantly lower figure compared to other countries, suggesting a possible underdiagnosis [104]. Additionally, it is important to note that studies on gigantism in Africa are limited, and none meet the inclusion criteria for this analysis, revealing a gap in the understanding of the disease in this region.

It is worth mentioning that although the Southeast Asia region showed the shortest average delay in diagnosis (2 years), the predominant presenting features (fatigue and heart failure) are more consistent with late-stage disease. This contradictory trend may be due to the low number of studies from that region; possibly, another factor involved could be publication bias, where more severe cases tend to be reported more often in the literature. These limitations highlight the need for more comprehensive, prospective data to validate temporal and clinical patterns of diagnosis across global regions.

On the other hand, reports regarding the prevalence of comorbidities related to acromegaly report a rate of hypertension and left ventricle hypertrophy of 20–50% and 25–85%, respectively [105,106]. A similar study reported a prevalence of diabetes of 55% [107]. Furthermore, a previous systematic review reported that cardiovascular comorbidities were the most prevalent at the time of diagnosis, representing 59% of comorbidities [8]. In contrast, in our review, we found that endocrine–metabolic alterations, a group that included diabetes, prediabetes, dyslipidemia, and polycystic ovarian syndrome, was the comorbidity with the highest prevalence (42.82%), followed by cardiovascular disease, which included myocardiopathy, heart failure, hypertension, hypertrophy, and vascular alterations, with 31.45%.

The diagnosis of acromegaly is compromised by several factors, including access to healthcare, available diagnostic tools (and their quality), as well as the incidence and prevalence of acromegaly cases depending on the location [108]. These directly affect the number of diagnosed patients, the delay in diagnosis, and the reporting of cases. Consequently, more cases and scientific articles are reported in countries of the European region and the Americas, while fewer cases are documented in the Eastern Mediterranean region [109]; a trend that was also shown in our review. The delay in diagnosing acromegaly and gigantism remains a significant challenge. Our review shows that the longest diagnostic delay occurred between 1963 and 1972, with an average of 10 years from symptom onset to diagnosis. This delay gradually decreased to an average of 5 years during the 1993–2002 period. However, from 2003 to 2012, there was an increase in diagnostic delay to almost 9 years, followed by a slight reduction to 7 years between 2013 and 2022. This trend suggests that, despite advances in awareness and diagnostic techniques, significant barriers to early detection still exist. These findings align with previous studies, which reported diagnostic delays of up to 14–15 years before the 1990s, with a gradual reduction over time, reaching an average of 6–7 years by the 2010s [110]. Nevertheless, some studies have reported shorter diagnostic times, ranging from 2.5 to 5.5 years [48,111]. This variability reflects differences in healthcare systems, access to diagnostic resources, and medical awareness of the disease, emphasizing the ongoing need to improve early diagnostic methods. Furthermore, it is important to remember that our study depends on what is reported on the literature, which is often cases with atypical presentation or that had some type of special characteristic to warrant publication; therefore, our data of the temporal trends should not be interpreted as a temporal analysis of actual diagnostic data as not all data is reported in the scientific literature and might be managed by the relevant health departments in each country.

Regarding diagnostic techniques, we tried to standardize the measurements for imaging diagnosis and included the greatest diameter of every tumor; we could not use other types of metrics such as the modified Knosp classification due to the scarcity of reporting [112]. We did not find any relationship between tumor size and year of publication (R = 0.0025 *p* = 0.989), tumor size and years of diagnostic delay (R = −0.1857, *p* = 0.462388), nor between years of diagnosis delay and year of publication (R = −0.0434, *p* = 0.794184). However, further statistical analysis between years of diagnostic delay and comorbidities should be performed in future studies. Regarding laboratory findings, IGF-1 values are preferred as the gold standard for diagnosis given that its values are not affected by glucose intake and its levels correlate with soft tissue enlargement and insulin resistance [113]. The normal range is dependent on the specific IGF-1 essay and should be calibrated accordingly [114]. To standardize these values, we used the IGF-1 index based on the range stated on each paper. In a paper that included 216 patients, the average IGF-1 was reported as 659 μg/L, with an IGF-1 index of 2.7 [48], similar to the accumulated values obtained in this review.

Another critical factor to consider in the diagnosis of acromegaly or gigantism is the presence of systemic barriers within both public and private healthcare systems. These include the high costs of medical care, limited access to advanced imaging studies such as magnetic resonance imaging (MRI), and inefficiencies in the referral process from primary care to specialist services. In the United States, it has been reported that healthcare costs related to patients with acromegaly amount to 6754 USD for hospitalizations and 6147 USD for medications, highlighting the considerable economic burden of this condition [115]. Therefore, the illness goes beyond physical manifestations and as physicians we must also consider quality of life (QoL). Certainly, previous studies have highlighted the importance of QoL as part of adequate management of acromegaly and gigantism; useful tools have been developed for this such as the AcroQoL questionnaire that considers the patients’ perspective of their symptoms and functional status at the time of diagnosis for decision making and for providing a patient-centered approach [116].

Moreover, recent studies illustrate how diagnostic evaluation of GH-secreting PitNETs has progressed in highly specialized centers, where classification now includes not only clinical features and imaging characteristics, but also intraoperative tumor consistency (categorized as soft, intermediate, or fibrous), histological subtype, and the expression of pituitary transcription factors such as PIT1 and SF1 [117,118]. While such approaches remain limited to specialized institutions, they open the door for more precise phenotypic characterization. However, comparative studies evaluating the global distribution of these transcriptional profiles and their correlation with clinical presentation are still lacking, which limits our broader understanding of the disease’s variability and epidemiology and should be considered as a future objective for research in this particular group of patients.

### Limitations

We would like to acknowledge several limitations to our systematic review. First, the quality and completeness of the data given in the main research naturally limits the results even with a thorough and methodically based search approach. Many contained case studies or case reports, which can be vulnerable to selection and publication bias, especially towards more severe or atypical presentations; furthermore, the low number of pediatric cases included in this study limits the generalization of our results in this population. Second, despite standardizing factors like tumor size and hormone levels, differences in reporting units and diagnostic thresholds among studies could have added variability. Third, the lack of uniform criteria for defining clinical signs, symptoms, and comorbidities limited our ability to perform meta-analyses and may have influenced the prevalence estimates. Fourth, although other areas, especially Africa and portions of Asia, were under-represented or not included at all, the literature disproportionately features data from particular areas (e.g., North America, Europe). This highlights a great discrepancy in the worldwide knowledge of GH-secreting PitNETs and restricts the generalizability of our geographical results. Ultimately, the retroactive character of the included data and the possible over-representation of unusual or late-presenting cases in the literature may cause diagnostic delay estimations and other temporal analyses to not reflect real-world diagnostic timeframes.

However, although the diagnosis of acromegaly and gigantism remains challenging, it is expected that future advances will improve this situation through the incorporation of new technologies, such as artificial intelligence and automated image analysis [119], capable of detecting subtle changes in the face and extremities, thereby facilitating early identification. In addition, the development of multinational registries and the implementation of telemedicine could broaden access to specialized screening, further shortening the gap between the onset and the diagnosis of this significant, life-altering disease.

## 5. Conclusions

Acromegaly and gigantism remain underdiagnosed, as showcased in our systematic evaluation of GH-secreting PitNETs, and are commonly discovered only following a protracted delay from symptom onset. Although developments in imaging, endocrinology, and clinical awareness have raised diagnosis rates over time, significant differences still exist worldwide, especially with relation to symptom identification and comorbidity profiles. The most often occurring clinical symptoms were facial and osteomuscular alterations; at the time of diagnosis, endocrine–metabolic and cardiovascular diseases predominated among the comorbidities. Particularly in under-represented areas, our results underline the need to raise awareness among doctors, consistent diagnosis procedures, and better access to healthcare resources. Prospective studies and population-level data should be the main emphasis of future studies to better define diagnostic trajectories, investigate predictive patterns, and lower the time to diagnosis for both gigantism and acromegaly.

## Figures and Tables

**Table 1:** Risk of Bias Assessment and Grading.

Design	Total Number of Patients	Risk of Bias (*n* of Articles)	References
Case report	7	Low (3)	[15,23,33]
		Moderate (2)	[30,38]
		High (2)	[37,91]
Case series	135	Low (51)	[10,11,12,13,14,18,19,22,25,26,27,28,29,32,33,34,36,42,45,49,51,53,55,56,58,59,62,63,64,65,70,71,72,74,77,79,80,81,82,83,84,87,89,90,93,94,95,99,100,101,102]
		Moderate (4)	[16,31,86,96]
		High (0)	
Case–control	406	Low (8)	[20,21,40,41,43,61,85,98]
		Moderate (3)	[24,39,67]
		High (0)	
Cross-sectional	1197	Low (2)	[46,92]
		Moderate (18)	[17,44,47,48,50,52,54,57,60,66,68,69,73,75,76,78,88,97]
		High (0)	

**Table 2:** Absolute frequencies of signs and symptoms, stratified by category and count in patients with acromegaly.

Category	Sign/Symptom 1 (n)	Sign/Symptom 2 (n)	Sign/Symptom 3 (n)	Sign/Symptom 4 (n)	Other Manifestations (n)
Facial changes	Changes in the jaw (211)	Frontal bossing (150)	Large nose (116)	Macroglossia (115)	384
Musculoskeletal	Large extremities (224)	Fatigue (172)	Arthralgia (142)	Back pain (52)	139
Cutaneous	Excessive sweating (109)	Thick skin (103)	Hirsutism (89)	Oily skin (81)	193
Weight	Central obesity (204)	Weight gain (125)	Overweight (52)	-	-
Neurological	Headache (202)	Memory loss (67)	Irritability (1)	Altered state of consciousness (1)	1
Respiratory	Snoring (79)	Changes in voice (21)	Dyspnea (16)	Cough (13)	-
Endocrine	Menstrual irregularity (33)	Galactorrhea (21)	Infertility (2)	Parotid gland enlargement (1)	2
Genitourinary	Libido alteration (16)	Erectile dysfunction (15)	Polyuria (6)	Urolithiasis (3)	1
Gastrointestinal	Abdominal pain (14)	Dysphagia (8)	Nausea/vomiting (3)	Hematochezia (1)	-
Visual	Visual field alteration (19)	Diplopia (1)	-	-	-
Psychological	Depression (10)	Behavioral changes (2)	-	-	-
Auditory	Hypoacusis (4)	-	-	-	-

**Table 3:** Absolute frequencies of signs and symptoms, stratified by category and count in patients with gigantism.

Category	Sign/Symptom 1 (n)	Sign/Symptom 2 (n)	Sign/Symptom 3 (n)	Sign/Symptom 4 (n)	Other Manifestations (n)
Facial changes	Changes in the jaw (6)	Large nose (3)	Large lips (3)	Macroglossia (1)	3
Musculoskeletal	Tall stature (7)	Large extremities (6)	Back pain (2)	Large hands (2)	6
Cutaneous	Soft tissue increase (3)	Thick skin (2)	Excessive sweating (1)	Acanthosis (1)	1
Weight	Central obesity (1)	-	-	-	-
Neurological	Headache (4)	Irritability (1)	-	-	-
Respiratory	Snoring (2)	Dyspnea (1)	-	-	-
Endocrine	Menstrual irregularity (1)	-	-	-	-
Genitourinary	Libido alteration (1)	Enuresis (1)	-	-	-
Gastrointestinal	Nausea/vomiting (2)	Constipation (1)	-	-	-
Visual	Visual field alteration (3)	Diplopia (1)	-	-	-
Psychological	Depression (1)	-	-	-	-

**Table 4:** Absolute frequencies of comorbid diagnosis, stratified by category and count in patients with acromegaly.

Category	Comorbidity 1 (n)	Comorbidity 2 (n)	Comorbidity 3 (n)	Comorbidity 4 (n)	Other Comorbidities (n)
Endocrine–metabolic	Diabetes (311)	Glucose intolerance (162)	Dyslipidemia (104)	PCOS (5)	0
Cardiovascular	Hypertension (327)	Cardiomyopathy (34)	Heart failure (26)	Impaired cardiac autonomic function (20)	21
Neoplasia/Hyperplasia	Colonic polyps (82)	Thyroid nodules (27)	Goiter (18)	Fibroadenoma (5)	5
Neurological	Carpal tunnel syndrome (57)	Peripheral neuropathy (43)	Generalized seizure (1)	-	0
Musculoskeletal	Gout (37)	Hernia (14)	Osteoarthritis (7)	Myopathy (2)	6
Respiratory	Obstructive sleep apnea (41)	-	-	-	0
Hematopoietic	Polycythemia (1)	-	-	-	0
Gastrointestinal	Hemorrhoids (1)	-	-	-	0

**Table 5:** Absolute frequencies of comorbid diagnosis, stratified by category and count in patients with gigantism.

Category	Comorbidity 1 (n)	Comorbidity 2 (n)
Endocrine–metabolic	Glucose intolerance (1)	Dyslipidemia (1)
Cardiovascular	Hypertension (1)	-
Neurological	Peripheral neuropathy (2)	-
Osteomuscular	Osteoarthritis (1)	Myopathy (1)

**Table 6:** Summary of the reported laboratory and imaging results, stratified by decade of reporting.

Decade	GH (μg/L)	IGF1 (μg/L)	IGF1 Index	Adenoma Size (mm)	Adenoma Range (mm)
1963–1972	27.8	Not reported	Not reported	Not reported	Not reported
1973–1982	71.6	Not reported	Not reported	Not reported	Not reported
1983–1992	39.4	Not reported	Not reported	Not reported	Not reported
1993–2002	35.9	667.8	1.3	13.7	4–43
2003–2012	16.0	663.3	3.3	19.5	11–28
2013–2022	14.8	639.3	2.7	16.1	2–28

## Data Availability

All data is available in the manuscript and Supplementary Materials.

## Supplementary Materials

The following supporting information can be downloaded at https://www.mdpi.com/article/10.3390/diagnostics15172137/s1; Section S1: Complete Search Criteria; Table S1: PRISMA 2020 checklist; Table S2: Data Extraction Table; Table S3: Signs and Symptoms—Absolute Frequencies; Table S4: Comorbidities—Absolute Frequencies.

## Abbreviations

The following abbreviations are used in this manuscript:

CENTRAL	Cochrane Central Register of Controlled Trials
GH	Growth hormone
IGF-1	Insulin-like growth factor 1
MeSH	Medical Subject Headings
NHLBI	National Heart, Lung, and Blood Institute
PitNET	Pituitary neuroendocrine tumor
PRISMA	Preferred Reporting Items for Systematic Reviews and Meta-Analyses
PROSPERO	International Prospective Register of Systematic Reviews
VHL	Virtual Health Library
WHO	World Health Organization
