# Supplementary material for: From Subtle Signs to Severe Sequelae—A Century of Symptomatology and Comorbidities in the Diagnosis of GH-Secreting Pituitary Neuroendocrine Tumors: A Systematic Review
Source: Diagnostics (Basel). 2025 Aug 24;15(17):2137. doi: 10.3390/diagnostics15172137 (PMC12428067; doi:10.3390/diagnostics15172137)
Supplement: Supplementary file 1 [file diagnostics-15-02137-s001.zip › diagnostics-3651590-supplementary.pdf]

Section S1: COMPLETE SEARCH CRITERIA

Search criteria for MEDLINE

((Growth Hormone-Secreting Pituitary Adenoma) OR (gigantism) OR (acromegaly) OR (Somatotropin Hypersecretion) OR ("Hyperpituitarism"[Mesh]) OR ("Acromegaly"[Mesh])) AND ((clinical features) OR (Signs) OR (Symptoms) OR (clinical manifestation) OR (Presenting signs) OR (Presenting symptoms) OR ("Signs and Symptoms"[Mesh])) NOT ((therapy) OR (treatment))

Search criteria for SCOPUS

(TITLE-ABS-KEY ((growth AND hormone-secreting AND pituitary AND adenoma) OR gigantism OR acromegaly OR (somatotropin AND hypersecretion)) AND TITLE-ABS-KEY ((signs OR symptoms) OR (presenting AND signs) OR (presenting AND symptoms) OR (clinical AND features)) AND NOT TITLE-ABS-KEY (therapy OR treatment))

Search criteria for CENTRAL

ID	Search	Hits
#1	MeSH descriptor: [Growth Hormone-Secreting Pituitary Adenoma] explode all trees	12
#2	gigantism	20
#3	acromegaly	511
#4	pituitary adenoma	298
#5	Somatotropin Hypersecretion	2
#6	MeSH descriptor: [Hyperpituitarism] explode all trees	431
#7	MeSH descriptor: [Acromegaly] explode all trees	243
#8	#1 OR #2 OR #3 OR #4 OR #5 OR #6 OR #7	905
#9	clinical features	20568
#10	Signs	48660
#11	Symptoms	179507
#12	Presenting signs	2128
#13	Presenting symptoms	5708
#14	clinical manifestation	3499
#15	MeSH descriptor: [Signs and Symptoms] explode all trees	152447
#16	#9 OR #10 OR #11 OR #12 OR #13 OR #14 OR #15	352908
#17	therapy	793309
#18	treatment	876970
#19	#17 OR #18	1136672
#20	#8 AND #16 NOT #19	22

Search criteria for the Virtual Health Library (VHL)

((((Growth Hormone-Secreting Pituitary Adenoma) OR (Adenoma hipofisario secretor de hormona de crecimiento)) OR ((acromegaly) OR (Acromegalia)) OR ((gigantism) OR (Gigantismo)) OR ((Somatotropin Hypersecretion) OR (Hipersecreción de somatotropina))) AND (((clinical features) OR (Caracteríticas clínicas)) OR ((Signs) or (Signos)) OR ((Symptoms) OR (Sintomas)) OR ((Presenting signs) OR (signos de presentación)) OR ((Presenting symptoms) OR (síntomas de presentación)) OR ((clinical manifestation) OR (Manifestación clínica)))) AND NOT (((therapy) OR (terapia)) OR ((treatment) OR (tratamiento)))

Table S1: PRISMA 2020 checklist.

Section and Topic	Item #	Checklist item	Location where item is reported
**TITLE**			
Title	1	Identify the report as a systematic review.	Pag. 1
**ABSTRACT**			
Abstract	2	See the PRISMA 2020 for Abstracts checklist.	Pag. 1
**INTRODUCTION**			
Rationale	3	Describe the rationale for the review in the context of existing knowledge.	Pag. 1-2
Objectives	4	Provide an explicit statement of the objective(s) or question(s) the review addresses.	Pag. 1-2
**METHODS**			
Eligibility criteria	5	Specify the inclusion and exclusion criteria for the review and how studies were grouped for the syntheses.	Pag. 2
Information sources	6	Specify all databases, registers, websites, organisations, reference lists and other sources searched or consulted to identify studies. Specify the date when each source was last searched or consulted.	Pag. 2-3
Search strategy	7	Present the full search strategies for all databases, registers and websites, including any filters and limits used.	Pag. 3 and Supplementary
Selection process	8	Specify the methods used to decide whether a study met the inclusion criteria of the review, including how many reviewers screened each record and each report retrieved, whether they worked independently, and if applicable, details of automation tools used in the process.	Pag. 3
Data collection process	9	Specify the methods used to collect data from reports, including how many reviewers collected data from each report, whether they worked independently, any processes for obtaining or confirming data from study investigators, and if applicable, details of automation tools used in the process.	Pag. 3
Data items	10a	List and define all outcomes for which data were sought. Specify whether all results that were compatible with each outcome domain in each study were sought (e.g. for all measures, time points, analyses), and if not, the methods used to decide which results to collect.	Pag. 3
	10b	List and define all other variables for which data were sought (e.g. participant and intervention characteristics, funding sources). Describe any assumptions made about any missing or unclear information.	Pag. 3
Study risk of bias assessment	11	Specify the methods used to assess risk of bias in the included studies, including details of the tool(s) used, how many reviewers assessed each study and whether they worked independently, and if applicable, details of automation tools used in the process.	Pag. 3
Effect measures	12	Specify for each outcome the effect measure(s) (e.g. risk ratio, mean difference) used in the synthesis or presentation of results.	Pag. 3-4
Synthesis methods	13a	Describe the processes used to decide which studies were eligible for each synthesis (e.g. tabulating the study intervention characteristics and comparing against the planned groups for each synthesis (item #5)).	Pag. 3-4
	13b	Describe any methods required to prepare the data for presentation or synthesis, such as handling of missing summary statistics, or data conversions.	Pag. 3-4

Section and Topic	Item #	Checklist item	Location where item is reported
	13c	Describe any methods used to tabulate or visually display results of individual studies and syntheses.	Pag. 3-4
	13d	Describe any methods used to synthesize results and provide a rationale for the choice(s). If meta-analysis was performed, describe the model(s), method(s) to identify the presence and extent of statistical heterogeneity, and software package(s) used.	NA
	13e	Describe any methods used to explore possible causes of heterogeneity among study results (e.g. subgroup analysis, meta-regression).	NA
	13f	Describe any sensitivity analyses conducted to assess robustness of the synthesized results.	NA
Reporting bias assessment	14	Describe any methods used to assess risk of bias due to missing results in a synthesis (arising from reporting biases).	Pag. 3-4
Certainty assessment	15	Describe any methods used to assess certainty (or confidence) in the body of evidence for an outcome.	Pag. 3-4
**RESULTS**			
Study selection	16a	Describe the results of the search and selection process, from the number of records identified in the search to the number of studies included in the review, ideally using a flow diagram.	Pag. 4
	16b	Cite studies that might appear to meet the inclusion criteria, but which were excluded, and explain why they were excluded.	Pag. 4
Study characteristics	17	Cite each included study and present its characteristics.	Pag- 5
Risk of bias in studies	18	Present assessments of risk of bias for each included study.	Pag. 5-6
Results of individual studies	19	For all outcomes, present, for each study: (a) summary statistics for each group (where appropriate) and (b) an effect estimate and its precision (e.g. confidence/credible interval), ideally using structured tables or plots.	Pag- 6-9
Results of syntheses	20a	For each synthesis, briefly summarise the characteristics and risk of bias among contributing studies.	Pag. 5
	20b	Present results of all statistical syntheses conducted. If meta-analysis was done, present for each the summary estimate and its precision (e.g. confidence/credible interval) and measures of statistical heterogeneity. If comparing groups, describe the direction of the effect.	Pag- 6-9
	20c	Present results of all investigations of possible causes of heterogeneity among study results.	NA
	20d	Present results of all sensitivity analyses conducted to assess the robustness of the synthesized results.	NA
Reporting biases	21	Present assessments of risk of bias due to missing results (arising from reporting biases) for each synthesis assessed.	Pag- 6-9
Certainty of evidence	22	Present assessments of certainty (or confidence) in the body of evidence for each outcome assessed.	Pag- 6-9
**DISCUSSION**			
Discussion	23a	Provide a general interpretation of the results in the context of other evidence.	Pag- 9-11
	23b	Discuss any limitations of the evidence included in the review.	Pag- 9-11
	23c	Discuss any limitations of the review processes used.	Pag- 9-11

Section and Topic	Item #	Checklist item	Location where item is reported
	23d	Discuss implications of the results for practice, policy, and future research.	Pag- 9-11
**OTHER INFORMATION**			
Registration and protocol	24a	Provide registration information for the review, including register name and registration number, or state that the review was not registered.	Pag. 2
	24b	Indicate where the review protocol can be accessed, or state that a protocol was not prepared.	Pag. 2
	24c	Describe and explain any amendments to information provided at registration or in the protocol.	Pag. 2
Support	25	Describe sources of financial or non-financial support for the review, and the role of the funders or sponsors in the review.	Pag. 12
Competing interests	26	Declare any competing interests of review authors.	Pag. 12
Availability of data, code and other materials	27	Report which of the following are publicly available and where they can be found: template data collection forms; data extracted from included studies; data used for all analyses; analytic code; any other materials used in the review.	Pag. 12

From: Page MJ, McKenzie JE, Bossuyt PM, Boutron I, Hoffmann TC, Mulrow CD, et al. The PRISMA 2020 statement: an updated guideline for reporting systematic reviews. BMJ 2021;372:n71. doi: 10.1136/bmj.n71. This work is licensed under CC BY 4.0. To view a copy of this license, visit https://creativecommons.org/licenses/by/4.0/

Table S2: DATA EXTRACTION TABLE

Author (year)	Country	Design	Bias	Total of patients	Sex: women	Sex: men	Age in years	Average age of onset of symptoms	Average age at diagnosis	Years of delay to diagnosis	IGF1 Index	GH (ug/L)	IGF1 (ug/L)	Tumor size (mm) greater diameter
Razvi (2007)	England	Case Series	Low	1	1	0	52	49	52	3	1.77	2,585	605,966	
Rogerio (2020)	Brazil	Case Series	Low	1	1	0	79	74	79	5	2.1	13,207	427,439	12.2
Avery (1973)	New Zealand	Case Series	Low	1	1	0	18	16	18	2		14.1		
Okada (1997)	Japan	Case Series	Low	1	1	0	28	20	28	8	1.89	21.9	828	10
Asai (1997)	Japan	Case Series	Low	1	1	0	58	44	58	14	1.09	2.2	420	
Cheah (1970)	China	Case Report	Low	1	0	1	51							
Wilson (1913)	England	Case Series	Moderate	1	0	1	27	16	27	11				
Gierach (2010)	Poland	Cross-Sectional	Moderate	86	48	38			48					

Triguero Veloz (2011)	Cuba	Case Series	Low	1	1	0	32		32			6		12.2
Good (1964)	United States	Case Series	Low	1	0	1	43	30	43	13				
Scarpa (2004)	Italy	Case-Control	Low	54	27	27	46.3	34.8	46.3	11.5				
Lusiani (1988)	Italy	Case-Control	Low	18	10	8	47	38	47	8.44		39.4		
O'Reilly (1997)	Ireland	Case Series	Low	1	0	1	60	50	60	10				
de Majo (1960)	Argentina	Case Report	Low	1	0	1	9	7	9	2				
Ilhan (2015)	Türkiye	Case-Control	Moderate	23	15	8	43.2		43.2		3.2	8.7	788.4	
Spence (1972)	United States	Case Series	Low	1	0	1	9.5	6	9.5	3.5		40		
Mbadugha (2020)	Japan	Case Series	Low	1	0	1	9	8.67	9	0.33	2.06	25	873	12
Al-Bedaia (2008)	Saudi Arabia	Case Series	Low	1	0	1	17	13	17	4		17,531		
Yerawar (2016)	India	Case Series	Low	1	0	1	41	40	41	1	3.59	48.1	958	21
Raju (2015)	England	Case Series	Low	1	0	1	43				1.63	0.7	470,012	
Jain (2012)	India	Case Report	Moderate	1	1	0	28	25	28	3	3.5	45.1	1344	
Mims (1974)	United States	Case Series	Moderate	3	1	2	50	44.6	50	5.6		6.72		
Lee (2015)	Korea	Case Series	Low	1	1	0	47		47		0.51		183.8	5
Vancil (1965)	United States	Case Report	Low	1	0	1	49	49	58	9				
Lisbona-Gil (2006)	Spain	Case Series	Low	1	1	0	56	47	56	9	1.71	17.9	833	
Castro (1999)	Netherlands	Case Series	Low	2	2	0	47	43	47	3.2		8.49	735	
Patel (2021)	United States	Case Series	Low	1	0	1	56		56				468	8
Das (2010)	England	Case Report	High	1	0	1	39							20
Gamal-AbdelNaser (2021)	Egypt	Case Report	Moderate	1	0	1	40	39	40	1			127	2
Bogazzi (2010)	Italy	Case-Control	Moderate	13	8	5	48					12	901	
Dural (2014)	United States	Case-Control	Low	20	11	9	45.7			5		9.7	608.5	
Freda (2003)	United States	Case-Control	Low	15	9	6	46.5	46.5				13.1	917.8	
Abraham (2004)	United States	Case Series	Low	1	0	1	26	22	26	4		12.5	1410	28
Ciulla (1999)	Italy	Case-Control	Low	10	4	6	39		39			22.1	99,596	

Kaji (2001)	Japan	Cross-Sectional	Moderate	26	13	13	51.8	51.8				35	676.2	
Sriphrapradang (2016)	Thailand	Case Series	Low	1	0	1	54	52	54	2				11
Guo (2018)	China	Cross-Sectional	Low	25	9	16	41.3			7.64		27.6	997.8	19.5
Espinosa-de-los-Monteros (2011)	United States	Cross-Sectional	Moderate	257	166	91					2	14		
Varlamov (2021)	United States	Cross-Sectional	Moderate	216	127	89	51				2.7	5.7	659	15
Milos (1996)	Sweden	Case Series	Low	1	0	1	37	33	37	4				10
Babic (2006)	United States	Cross-Sectional	Moderate	30	18	12	46.1	37.5	46.1	8.6		44.74	690.3	
Rioperez (1981)	Germany	Case Series	Low	1	1	0	45	33	45	12		16		
Akoglu (2013)	Türkiye	Cross-Sectional	Moderate	49	27	22	45.5	36.5	45.2	8.7				
Chentli (2015)	France	Case Series	Low	5	1	4	33					1.88	1279	23
Montefusco (2010)	Italy	Cross-Sectional	Moderate	76	46	30			44.5		7.7	14.8		
Singla (2021)	India	Case Series	Low	1	1	0	36		36			22	1402	20
Moore (2000)	England	Case Series	Low	2	1	1	58	56.75	58	1.25			701	
Benfante (2015)	United States	Cross-Sectional	Moderate	3	1	2	46.67		46.67	1				
Rowe (1934)	United States	Case Series	Low	1	0	1	26	8	26	18				
Zafar (2004)	United States	Case Series	Low	1	1	0	32	26	32	6				
Duru (2016)	Türkiye	Cross-Sectional	Moderate	29	18	11	51.3		51.34					
Leon-Carrion (2010)	Spain	Case-Control	Low	16	12	4		38.5				19,557	889,481	21
Tejera Pérez (2022)	Spain	Case Series	Low	1	1	0	46	36	46	10		40	944	20
Suzuki (2014)	Japan	Case Series	Low	1	0	1	76					47.7	1204	
Malicka (2011)	Poland	Case Series	Low	2	2	0	38.67	32	38	6				14.5

Tiryakioğlu (2004)	Türkiye	Case Series	Low	3	2	1	29.67	22	24	2				
Dogansen (2018)	United States	Cross-Sectional	Moderate	36	36	0	34	29.6	34	4.25		25.15	757.5	16
Inayet (2020)	England	Case-Control	Moderate	50	26	24	43		32.44	10.56				
Colao (2002)	Italy	Cross-Sectional	Moderate	151	79	72			50.3		1.28	36.5	708.3	13.7
Çapoglu (2002)	Türkiye	Cross-Sectional	Moderate	1	0	1	35		34			17.4	0.198	
Saeki (2000)	Japan	Case Series	Low	1	0	1	58					7.3	10.72	4
Jamjoom (1995)	Saudi Arabia	Case Series	Low	18	4	14	37.2	31.8	37.2	5.4		48.6		
Uchida (2003)	Japan	Case Series	Low	1	1	0	40	36	40	4	2.11	78.94	923	
Sumbul (2019)	Türkiye	Cross-Sectional	Moderate	57	22	35	41.8		41.8			7.33	3,278	
Asa (1980)	Canada	Case Series	Low	1	1	0	26							
Klijn (1980)	Netherlands	Cross-Sectional	Moderate	44	21	23						69.5		
Foltyn (2008)	Poland	Cross-Sectional	Moderate	40	24	16	51.36		51.36	8.36				
De Menis (2002)	Italy	Case Series	Low	2	1	1	18	17	18	1	3.98	21.5	1512	9
Yoshida (2013)	Japan	Cross-Sectional	Moderate	9	2	7	38.8		38.8			155	982	
Subramnaian (2021)	India	Case Series	Low	1	0	1	56	55.75	56	0.25		11.2	622.2	
Elarabi (2018)	Qatar	Case Series	Low	1	0	1	21					28.6	908.1	
Lopis (1968)	South Africa	Case Series	Low	1	0	1	12	10	12	2				
Nagulesparen (1976)	England	Case Series	Low	18	8	10	54.5	45	35.5	9.5		93.72		
Ferrer García (2007)	Spain	Case Series	Low	1	1	0	28	23	28	5		2.39	800	20
Lewis (1972)	England	Case Series	Low	1	0	1	26	5	26	21		15.51		
Arikan (2010)	Türkiye	Case-Control	Low	22	9	13	38.85		38.85					
Howard (1965)	United States	Case Series	Moderate	1	1	0	30		30					

Mandel (2020)	United States	Case Series	Low	1	0	1	49	47	49	2				
Low (1974)	England	Cross-Sectional	Moderate	2	0	2	51	42	51	9				
Pokhrel (2021)	Nepal	Case Series	Low	1	0	1	26		26					
Roelfsema (1998)	Netherlands	Case Series	Low	2	0	2	42.5	39	42.5	4.5		5.92	734,152	
Mantri (2016)	United States	Case Report	High	1	0	1	35	20	35	15	3.42	17.8	771	3
Hashim (2022)	India	Cross-Sectional	Low	35	18	17	39.77	34.34		5.43		24.77	597.7	
Heireman (2011)	India	Case Series	Low	1	0	1	45	44	45	1				12
Agrawal (2013)	England	Case Series	Low	1	0	1	36	35	36	1			1600	
Tran (2002)	Australia	Case Series	Low	1	0	1	38	37	38	1	1.59	0.658	52,051	
Arya (1997)	India	Case Series	Moderate	34	15	19		32.3	37.49	5.19				
Guo (2018)	United States	Cross-Sectional	Moderate	25	9	16	41.3		33.66	7.64		27.6	997.8	19.5
Gonzalez (2017)	Mexico	Case-Control	Low	165	97	68	47.5							
Imran (2018)	England	Case Series	Low	1	0	1	15	11	15	4		13.4	1600	4
Zangeneh (2002)	United States	Case Series	Low	1	0	1	45	44	45	1	3.75	160	1350	43
Bolton (2018)	United States	Case Series	Low	1	0	1	56	40	56	16				
Muthusamy (2010)	United States	Case Series	Low	1	0	1	58				2.75	25.3	747	11

**Author (year)**	**Tegumentary**	**Visual Field**	**Auditory**	**Psychological**	**Gastrointestinal**	**Genitourinary**	**Osteomuscular**	**Neurological**	**Weight**	**Facial Changes**	**Respiratory**	**Endocrine**
Razvi (2007)	1	0	0	0	0	0	1	1	0	2	1	0
Rogerio (2020)	0	0	0	0	0	0	1	0	0	3	0	0
Avery (1973)	1	1	0	1	2	1	1	1	1	0	0	1
Okada (1997)	1	0	0	0	0	0	1	0	0	4	1	0
Asai (1997)	1	0	0	0	0	0	2	1	0	3	0	0
Cheah (1970)	2	0	0	0	0	0	4	1	0	3	0	0
Wilson (1913)	2	1	0	0	0	0	3	1	0	1	0	0

Gierach (2010)	0	0	0	0	0	0	0	0	0	0	0	0
Triguero Veloz (2011)	3	0	0	0	0	0	3	0	0	4	0	1
Good (1964)	3	0	0	0	0	0	5	0	1	2	1	0
Scarpa (2004)	0	0	0	0	7	0	39	0	0	0	0	0
Lusiani (1988)	0	0	0	0	0	0	0	0	0	0	0	0
O'Reilly (1997)	1	0	0	0	0	0	1	0	0	3	0	0
de Majo (1960)	1	0	0	0	0	0	3	2	0	5	0	0
Ilhan (2015)	0	0	0	0	0	0	0	0	0	0	0	0
Spence (1972)	0	1	0	0	1	1	1	0	0	2	0	0
Mbadugha (2020)	0	0	0	0	0	0	2	1	0	2	1	0
Al-Bedaia (2008)	4	0	0	0	0	0	1	0	0	3	0	0
Yerawar (2016)	1	0	0	0	0	0	1	0	0	3	0	0
Raju (2015)	0	0	0	0	0	2	0	0	1	0	0	0
Jain (2012)	4	0	0	0	1	0	0	0	2	2	0	0
Mims (1974)	2	0	0	0	0	1	5	0	1	4	0	0
Lee (2015)	3	0	0	0	0	0	2	0	1	1	1	0
Vancil (1965)	0	0	0	0	0	1	1	0	0	3	0	0
Lisbona-Gil (2006)	0	0	0	0	0	0	1	0	0	2	0	0
Castro (1999)	1	0	0	0	0	0	1	1	1	0	0	1
Patel (2021)	0	0	0	0	0	0	1	0	0	2	0	0
Das (2010)	0	0	0	0	0	0	0	1	0	0	0	0
Gamal-AbdelNaser (2021)	0	1	0	0	0	0	3	0	0	0	0	0
Bogazzi (2010)	0	0	0	0	0	0	0	0	0	0	13	0
Dural (2014)	0	0	0	0	0	0	0	0	0	0	0	0
Freda (2003)	8	0	0	0	0	2	12	3	0	12	7	6
Abraham (2004)	1	0	0	0	0	2	1	0	0	1	0	0

Ciulla (1999)	0	0	0	0	0	0	0	0	0	0	0	0
Kaji (2001)	0	0	0	0	0	0	26	0	26	0	0	0
Sriphrapradang (2016)	1	0	0	0	0	0	0	0	0	2	0	0
Guo (2018)	0	0	0	0	0	0	0	0	0	0	0	0
Espinosa-de-los-Monteros (2011)	0	0	0	0	0	0	0	0	124,902	0	0	0
Varlamov (2021)	355.8	0	0	0	0	0	416	192.1	131.98	622.8	71.1	0
Milos (1996)	0	0	0	0	0	0	0	1	0	2	0	0
Babic (2006)	0	0	4	0	0	0	0	0	0	0	0	0
Rioperez (1981)	0	0	4	0	0	0	1	1	0	0	0	0
Akoglu (2013)	43	0	0	0	0	0	0	20	0	150	0	0
Chentli (2015)	1	0	0	0	0	0	0	0	0	0	0	0
Montefusco (2010)	0	0	0	0	0	0	0	0	0	0	0	0
Singla (2021)	0	0	0	0	0	0	1	0	0	3	0	0
Moore (2000)	0	0	0	0	1	0	1	0	0	9	1	0
Benfante (2015)	0	0	0	0	0	0	0	0	0	0	0	0
Rowe (1934)	0	0	0	0	0	0	3	0	0	1	0	0
Zafar (2004)	1	0	0	0	0	0	0	1	1	1	1	0
Duru (2016)	0	0	0	0	0	0	0	0	0	0	0	0
Leon-Carrion (2010)	0	1	0	10	0	0	0	0	0	0	0	0
Tejera Pérez (2022)	0	1	0	0	0	0	2	0	0	0	0	2
Suzuki (2014)	2	0	0	0	0	0	3	0	0	0	0	0
Malicka (2011)	4	0	0	0	0	0	5	1	0	4	1	0
Tiryakioğlu (2004)	2	0	0	0	0	0	3	3	0	0	0	2
Dogansen (2018)	16	0	0	0	0	2	0	0	0	0	0	18
Inayet (2020)	0	0	0	0	0	0	0	0	0	0	0	0

Colao (2002)	0	0	0	0	0	0	0	0	0	0	0	0
Çapoglu (2002)	1	0	0	0	0	0	0	0	0	1	0	0
Saeki (2000)	0	0	0	0	0	0	1	0	0	0	0	0
Jamjoom (1995)	5	5	0	2	0	10	16	11	5	54	2	2
Uchida (2003)	0	0	0	0	0	0	1	1	0	1	0	1
Sumbul (2019)	0	0	0	0	0	0	0	0	0	0	0	0
Asa (1980)	0	0	0	0	0	0	1	1	0	1	0	1
Klijn (1980)	83	6	0	0	14	21	92	27	40	0	0	21
Foltyn (2008)	0	0	0	0	0	0	0	0	19	0	0	0
De Menis (2002)	0	0	0	0	0	0	1	0	0	0	0	1
Yoshida (2013)	0	0	0	0	0	0	0	0	0	0	0	0
Subramnaian (2021)	2	0	0	0	0	0	1	0	0	2	0	0
Elarabi (2018)	0	1	0	0	0	0	2	0	0	4	1	0
Lopis (1968)	0	0	0	0	0	0	4	0	0	1	0	0
Nagulesparen (1976)	0	0	0	0	0	0	0	0	0	0	0	0
Ferrer García (2007)	0	0	0	0	0	0	1	1	1	0	0	0
Lewis (1972)	0	0	0	0	0	0	2	0	0	1	0	0
Arikan (2010)	0	0	0	0	0	0	0	0	22	0	0	0
Howard (1965)	0	0	0	0	0	0	2	0	1	2	0	1
Mandel (2020)	0	0	0	0	0	0	0	0	0	7	0	1
Low (1974)	0	0	0	0	0	0	0	0	0	0	0	0
Pokhrel (2021)	1	1	0	0	1	0	2	1	0	5	0	0
Roelfsema (1998)	2	1	0	0	2	0	3	2	0	1	0	1
Mantri (2016)	2	0	0	0	0	0	4	0	0	1	1	0
Hashim (2022)	0	0	0	0	0	0	17	0	0	0	25	0
Heireman (2011)	1	1	0	0	0	0	1	0	0	1	0	0

Agrawal (2013)	0	0	0	0	0	0	2	0	0	3	0	0
Tran (2002)	0	1	0	0	0	0	2	0	2	2	0	0
Arya (1997)	18	0	0	0	0	0	34	0	0	34	0	0
Guo (2018)	0	0	0	0	0	0	0	0	0	0	0	0
Gonzalez (2017)	0	0	0	0	0	0	0	0	0	0	0	0
Imran (2018)	0	1	0	0	0	0	2	0	0	0	2	0
Zangeneh (2002)	2	1	0	0	0	0	2	1	0	4	1	0
Bolton (2018)	0	0	0	0	0	0	1	0	0	4	0	0
Muthusamy (2010)	0	0	0	0	0	0	1	0	0	1	1	0

Table S3: Signs and Symptoms – Absolute Frequencies

Category	Sign/Symptom	Patients
Facial Changes	Changes in the jaw (mandible)	217
Facial Changes	Frontal bossing	150
Facial Changes	Large nose	119
Facial Changes	Macroglossia (enlarged tongue)	116
Facial Changes	Large lips	111
Facial Changes	Coarse facial features	103
Facial Changes	Diastema (gap between teeth)	83
Facial Changes	Round face	57
Facial Changes	Changes in facial appearance	34

Facial Changes	Enlarged sinuses	1
Facial Changes	Gingival enlargement	1
Osteomuscular	Large extremities	230
Osteomuscular	Fatigue	173
Osteomuscular	Arthralgia	142
Osteomuscular	Back pain	54
Osteomuscular	Neck pain	41
Osteomuscular	Large feet	36
Osteomuscular	Lean body mass	26
Osteomuscular	Large hands	22
Osteomuscular	Tall stature	11
Osteomuscular	Weakness	6
Osteomuscular	Skull thickening	5
Osteomuscular	Thick heel pad	2
Osteomuscular	Bony swellings	2
Osteomuscular	Spade-like hands	2
Tegumentary	Excessive sweating	110
Tegumentary	Thick skin	105
Tegumentary	Hirsutism	89
Tegumentary	Oily skin	81
Tegumentary	Skin tags	78
Tegumentary	Soft tissue increase	54
Tegumentary	Hand edema	46
Tegumentary	Acne	8
Tegumentary	Acanthosis	6
Tegumentary	Peripheral edema	3
Tegumentary	Cutis verticis gyrata	2
Tegumentary	Non-specific skin changes	1

Weight	Central obesity	205
Weight	Weight gain	125
Weight	Overweight	52
Neurological	Headache	206
Neurological	Memory loss	67
Neurological	Irritability	2
Neurological	Altered state of consciousness	1
Neurological	Corpus callosum agenesia and right cerebral hemiatrophy	1
Respiratory	Snoring	81
Respiratory	Changes in voice	21
Respiratory	Dyspnea (shortness of breath)	17
Respiratory	Cough	13
Endocrine	Menstrual irregularity	34
Endocrine	Galactorrhea	21
Endocrine	Infertility	2
Endocrine	Parotid gland enlargement	1
Endocrine	Heat intolerance	1
Endocrine	Gynecomastia	1
Genitourinary	Libido alteration	17
Genitourinary	Erectile dysfunction	15
Genitourinary	Polyuria	6
Genitourinary	Urolithiasis	3
Genitourinary	Enuresis	1
Genitourinary	Enlarged testicles	1
Gastrointestinal	Abdominal pain	14
Gastrointestinal	Dysphagia	8
Gastrointestinal	Nausea/vomiting	5

Gastrointestinal	Constipation	1
Gastrointestinal	Hematochezia	1
Visual	Visual field alteration	22
Visual	Diplopia	2
Psychological	Depression	11
Psychological	Behavioral changes	2
Auditory	Hypoacusis	4

Table S4: Comorbidities – Absolute Frequencies.

**Category**	**Comorbidity**	**Patients**
Endocrine metabolic	Diabetes	311
Endocrine metabolic	Glucose intolerance	163
Endocrine metabolic	Dyslipidemia	105
Endocrine metabolic	Polycystic ovary syndrome	5
Cardiovascular disease	Hypertension	328
Cardiovascular disease	Cardiomyopathy	34
Cardiovascular disease	Heart failure	26
Cardiovascular disease	Impaired cardiac autonomic functions	20
Cardiovascular disease	Coronary artery disease	10
Cardiovascular disease	Left ventricular hypertrophy	8
Cardiovascular disease	Arrhythmia	1
Cardiovascular disease	Vascular disease, tortuous aorta	1

Cardiovascular disease	Pulmonary embolism	1
Neoplasia/ hyperplasia	Colonic polyps	82
Neoplasia/ hyperplasia	Thyroid nodules	27
Neoplasia/ hyperplasia	Goiter	18
Neoplasia/ hyperplasia	Fibroadenoma (breast)	5
Neoplasia/ hyperplasia	Organomegaly	2
Neoplasia/ hyperplasia	Gastric cancer/neoplasia	1
Neoplasia/ hyperplasia	Basal cell carcinoma	1
Neoplasia/ hyperplasia	Pancreatic cyst	1
Neurologic	Carpal tunnel syndrome	57
Neurologic	Peripheral neuropathy	45
Neurologic	Generalized seizure	1
Osteomuscular	Gout	37
Osteomuscular	Inguinal/abdominal hernia	14
Osteomuscular	Osteoarthritis	8
Osteomuscular	Myopathy	3
Osteomuscular	Osteoporosis	3
Osteomuscular	Decreased bone mass	2
Osteomuscular	Vertebral fractures	1
Respiratory	Obstructive sleep apnea	41
Hematopoyetic	Polycythemia	1
Gastrointestinal	Hemorrhoids	1

References

1. Razvi, S.; Perros, P. A 52-Year-Old Female with a Hoarse Voice and Tingling in the Hand. *PLoS Med.* **2007**, *4*, e29. https://doi.org/10.1371/journal.pmed.0040029.
2. Rogerio, F.; Tamanini, J.V.G.; Gerson, G.; Haiter, T.C.; Queiroz, L.D.S.; Fabbro, M.D. A 79-Year-Old Woman with Headache and Acromegaly. *Brain Pathol.* **2020**, *30*, 407–408. https://doi.org/10.1111/bpa.12815.

3. Avery, T.L. A Case of Acromegaly and Gigantism with Depression. *Br. J. Psychiatry J. Ment. Sci.* **1973**, *122*, 599–600. https://doi.org/10.1192/bjp.122.5.599.
4. Okada, Y.; Morimoto, I.; Ejima, K.; Kashimura, M.; Yoshida, K.; Fujihira, T.; Eto, S. A Case of Active Acromegalic Woman with a Marked Increase in Serum Insulin-like Growth Factor-1 Levels after Delivery. *Endocr. J.* **1997**, *44*, 117–120. https://doi.org/10.1507/endocrj.44.117.
5. Asai, K.; Shimoyama, S.; Sanno, N.; Kaminishi, M.; Oohara, T. A rare case of gastric cancer in an acromegalic patient. *J. Gastroenterol.* **1997**, *32*, 528–532. https://doi.org/10.1007/bf02934094.
6. Cheah, J.S. A Singapore giant (gigantism with acromegaly): Untreated with long survival. *Med. J. Aust.* **1970**, *2*, 1250–1252. https://doi.org/10.5694/j.1326-5377.1970.tb63455.x.
7. Wilson, S.A.K. A Typical Case of Acromegaly, with Signs of Regression. *Proc. R. Soc. Med.* **1913**, *6*, 22–23. https://doi.org/10.1177/003591571300600846.
8. Gierach, M.; Gierach, J.; Pujanek, M.; Skowrońska, A.; Rutkowska, E.; Junik, R. Aberrations in carbohydrate metabolism in patients with diagnosed acromegaly, hospitalized in the Endocrinology and Diabetology Department of Collegium Medicum University of Nicolaus Copernicus in Bydgoszcz in the years 2001–2009. *Endokrynol. Pol.* **2010**, *61*, 260–263.
9. Triguero Veloz, M.N.; Pérez, J.C.G. Acromegalia. Presentación de un caso. *Rev. Cienc. Médicas Pinar Río* **2011**, *15*, 238–245.
10. Good, A.E. Acromegalic arthropathy. A case report. *Arthritis Rheum.* **1964**, *7*, 65–74. https://doi.org/10.1002/art.1780070109.
11. Scarpa, R.; De Brasi, D.; Pivonello, R.; Marzullo, P.; Manguso, F.; Sodano, A.; Oriente, P.; Lombardi, G.; Colao, A. Acromegalic Axial Arthropathy: A Clinical Case-Control Study. *J. Clin. Endocrinol. Metab.* **2004**, *89*, 598–603. https://doi.org/10.1210/jc.2003-031283.
12. Lusiani, L.; Ronsisvalle, G.; Visonà, A.; Castellani, V.; Bonanome, A.; Pagnan, A.; Facchin, F.; Sicolo, N.; Federspil, G. Acromegalic cardiomyopathy. An echocardiography study. *J. Endocrinol. Investig.* **1988**, *11*, 159–164. https://doi.org/10.1007/bf03350125.
13. O'REilly, F.M.; Sliney, I.; Frcpi, S.O. Acromegaly and Cutis Verticis Gyrata. *J. R. Soc. Med.* **1997**, *90*, 79. https://doi.org/10.1177/014107689709000206.
14. de Majo, S.F.; Oñativia, A. Acromegaly and gigantism in a boy: Comparison with 3 overgrown nonacromegalic children. *J. Pediatr.* **1960**, *57*, 382–390. https://doi.org/10.1016/s0022-3476(60)80246-6.
15. Ilhan, M.; Danalioglu, A.; Turgut, S.; Karaman, O.; Arabaci, E.; Tasan, E. Acromegaly Can Be Associated with Impairment of LES Relaxation in the Oesophagus. *Endokrynol. Polska* **2015**, *66*, 308–312. https://doi.org/10.5603/EP.2015.0039.
16. Spence, H.J.; Trias, E.P.; Raiti, S. Acromegaly in a 9 and One-Half-Year-Old Boy. Pituitary Function Studies before and after Surgery. *Am. J. Dis. Child.* **1972**, *123*, 504–506. https://doi.org/10.1001/archpedi.1972.02110110132018.
17. Mbadugha, T.; Ogiwara, T.; Nagm, A.; Hasegawa, T.; Kamiya, K.; Matsumoto, Y.; Kobayashi, M.; Hongo, K. Acromegaly in preadolescence: A case report of a 9-year-old boy with acromegaly. *Surg. Neurol. Int.* **2020**, *11*, 61. https://doi.org/10.25259/sni_474_2019.
18. Al-Bedaia, M.; Al-Khenaizan, S. Acromegaly presenting as cutis verticis gyrata. *Int. J. Dermatol.* **2008**, *47*, 164. https://doi.org/10.1111/j.1365-4632.2008.03350.x.
19. Yerawar, C.; Bandgar, T.; Lila, A.; Shah, N.S. Acromegaly presenting as cutis verticis gyrata. *QJM Int. J. Med.* **2016**, *109*, 423. https://doi.org/10.1093/qjmed/hcw020.
20. Raju, J.A.; Shipman, K.E.; Inglis, J.A.; Gama, R. Acromegaly Presenting as Erectile Dysfunction: Case Reports and Review of the Literature. *Rev. Urol.* **2015**, *17*, 246–249.
21. Jain, R.; Dutta, D.; Shivaprasad, K.; Maisnam, I.; Ghosh, S.; Mukhopadhyay, S.; Chowdhury, S. Acromegaly presenting as hirsuitism: Uncommon sinister aetiology of a common clinical sign. *Indian J. Endocrinol. Metab.* **2012**, *16*, 297–299. https://doi.org/10.4103/2230-8210.104066.
22. Mims, R.B.; Bethune, J.E. Acromegaly with Normal Fasting Growth Hormone Concentrations but Abnormal Growth Hormone Regulation. *Ann. Intern. Med.* **1974**, *81*, 781–784. https://doi.org/10.7326/0003-4819-81-6-781.

23. Lee, H.M.; Lee, S.H.; Yang, I.-H.; Hwang, I.K.; Hwang, Y.C.; Ahn, K.J.; Chung, H.Y.; Hwang, H.-J.; Jeong, I.-K. Acromegaly with Normal Insulin-Like Growth Factor-1 Levels and Congestive Heart Failure as the First Clinical Manifestation. *Endocrinol. Metab.* **2015**, *30*, 395–401. https://doi.org/10.3803/enm.2015.30.3.395.
24. Vancil, M.; Locke, W. Acromegaly, hyperparathyroidism, and probable mammary fibroadenoma in a man. *Am. J. Surg.* **1965**, *110*, 495–497. https://doi.org/10.1016/0002-9610(65)90100-5.
25. Lisbona-Gil, A..; Robledo, M.; Riestra, A.F.; Rodríguez, C.A. Acromegalia, hiperparatiroidismo primario y feocromocitoma. *Endocrinol. Nutr.* **2006**, *53*, 382–386. https://doi.org/10.1016/s1575-0922(06)71119-6.
26. Castro Cabezas, M.; Zelissen, P.M.; Jansen, G.H.; Van Gils, A.P.; Koppeschaar, H.P. Acromegaly: Report of two patients with an unusual presentation. *Neth. J. Med.* **1999**, *54*, 163–166. https://doi.org/10.1016/s0300-2977(99)00003-0.
27. Patel, S.; Minish, J.M.; Keeley, E.C. Acromegaly-Induced Cardiomyopathy Masquerading as an Acute Coronary Syndrome. *Am. J. Med.* **2021**, *134*, e51–e52. https://doi.org/10.1016/j.amjmed.2020.05.045.
28. Das, S.; Bhansali, A.; Dutta, P.; Khandelwal, N.; Upreti, V.; Santosh, R. An unusual association of corpus callosum agenesis in a patient with acromegaly. *BMJ Case Rep.* **2010**, *2010*, bcr0120102625. https://doi.org/10.1136/bcr.01.2010.2625.
29. Gamal-AbdelNaser, A. An Unusual Early Oral Presentation of Acromegaly: A Case Report. *Arch. Orofac. Sci.* **2021**, *16*, 253–258. https://doi.org/10.21315/aos2021.16.2.14.
30. Bogazzi, F.; Nacci, A.; Campomori, A.; La Vela, R.; Rossi, G.; Lombardi, M.; Fattori, B.; Bartalena, L.; Ursino, F.; Martino, E. Analysis of voice in patients with untreated active acromegaly. *J. Endocrinol. Investig.* **2010**, *33*, 178–185. https://doi.org/10.1007/bf03346578.
31. Dural, M.; Kabakcı, G.; Çınar, N.; Erbaş, T.; Canpolat, U.; Gürses, K.M.; Tokgözoğlu, L.; Oto, A.; Kaya, E.B.; Yorgun, H.; et al. Assessment of cardiac autonomic functions by heart rate recovery, heart rate variability and QT dynamicity parameters in patients with acromegaly. *Pituitary* **2014**, *17*, 163–170. https://doi.org/10.1007/s11102-013-0482-4.
32. Freda, P.U.; Reyes, C.M.; Nuruzzaman, A.T.; Sundeen, R.E.; Bruce, J.N. Basal and Glucose-Suppressed GH Levels Less Than 1 μg/L in Newly Diagnosed Acromegaly. *Pituitary* **2003**, *6*, 175–180. https://doi.org/10.1023/b:pitu.0000023424.72021.e2.
33. Abraham, D.; Couldwell, W. Bilateral testicular enlargement and seminoma in a patient with acromegaly. *Br. J. Neurosurg.* **2004**, *18*, 629–631. https://doi.org/10.1080/02688690400022797.
34. Ciulla, M.; Arosio, M.; Barelli, M.V.; Paliotti, R.; Porretti, S.; Valentini, P.; Tortora, G.; Buonamici, V.; Moraschi, A.; Cappiello, V.; et al. Blood pressure-independent cardiac hypertrophy in acromegalic patients. *J. Hypertens.* **1999**, *17*, 1965–1969. https://doi.org/10.1097/00004872-199917121-00028.
35. Kaji, H.; Sugimoto, T.; Nakaoka, D.; Okimura, Y.; Kaji, H.; Abe, H.; Chihara, K. Bone metabolism and body composition in Japanese patients with active acromegaly. *Clin. Endocrinol.* **2001**, *55*, 175–181. https://doi.org/10.1046/j.1365-2265.2001.01280.x.
36. Sriphrapradang, C.; Ngarmukos, C. Bulldog scalp. *Clevel. Clin. J. Med.* **2016**, *83*, 90–91. https://doi.org/10.3949/ccjm.83a.15019.
37. Guo, X.; Gao, L.; Zhao, Y.; Wang, M.; Jiang, B.; Wang, Q.; Wang, Z.; Liu, X.; Feng, M.; Wang, R.; et al. Characteristics of the upper respiratory tract in patients with acromegaly and correlations with obstructive sleep apnoea/hypopnea syndrome. *Sleep Med.* **2018**, *48*, 27–34. https://doi.org/10.1016/j.sleep.2018.04.011.
38. Espinosa-De-Los-Monteros, A.L.; González, B.; Vargas, G.; Sosa, E.; Mercado, M. Clinical and biochemical characteristics of acromegalic patients with different abnormalities in glucose metabolism. *Pituitary* **2011**, *14*, 231–235. https://doi.org/10.1007/s11102-010-0284-x.

39. Varlamov, E.V.; Niculescu, D.A.; Banskota, S.; Galoiu, S.A.; Poiana, C.; Fleseriu, M. Clinical features and complications of acromegaly at diagnosis are not all the same: Data from two large referral centers. *Endocr. Connect.* **2021**, *10*, 731–741. https://doi.org/10.1530/ec-21-0035.
40. Milos, P.; Havelius, U.; Hindfelt, B. Clusterlike Headache in a Patient with a Pituitary Adenoma. With a Review of Literature. *Headache J. Head Face Pain* **1996**, *36*, 184–188. https://doi.org/10.1046/j.1526-4610.1996.3603184.x.
41. Babic, B.B.; Petakov, M.S.; Djukic, V.B.; Ognjanovic, S.I.; Arsovic, N.A.; Isailovic, T.V.; Milovanovic, J.D.; Macut, D.; Damjanovic, S.S. Conductive Hearing Loss in Patients with Active Acromegaly. *Otol. Neurotol.* **2006**, *27*, 865–870. https://doi.org/10.1097/01.mao.0000201429.57746.1b.
42. Rioperez, E.; Botella, J.; Valdivieso, L.; Ballesteros, D.; Diez, L.; Navas, J. Conn's Syndrome in a Patient with Acromegaly. *Horm. Metab. Res.* **1981**, *13*, 186–187. https://doi.org/10.1055/s-2007-1019216.
43. Akoglu, G.; Metin, A.; Emre, S.; Ersoy, R.; Cakir, B. Cutaneous findings in patients with acromegaly. *ACTA Dermatovenerol. Croat.* **2013**, *21*, 224–229.
44. Chentli, F.; Terki, B.; Azzoug, S. Cutis verticis gyrata and acromegaly. *Presse Méd.* **2015**, *44*, 958–961. https://doi.org/10.1016/j.lpm.2015.03.019.
45. Montefusco, L.; Filopanti, M.; Ronchi, C.L.; Olgiati, L.; La-Porta, C.; Losa, M.; Epaminonda, P.; Coletti, F.; Beck-Peccoz, P.; Spada, A.; et al. d3-Growth hormone receptor polymorphism in acromegaly: Effects on metabolic phenotype. *Clin. Endocrinol.* **2010**, *72*, 661–667. https://doi.org/10.1111/j.1365-2265.2009.03703.x.
46. Singla, M.; Saini, J.K. Diabetes Mellitus of Pituitary Origin: A Case Report. *Eur. Endocrinol.* **2021**, *17*, 68. https://doi.org/10.17925/ee.2021.17.1.68.
47. Moore, M.; Spitteri-Staines, K.; Felix, D.; Critchlow, H. Diagnosis of Acromegaly in Orofacial Pain: Two Case Reports. *Dent. Updat.* **2000**, *27*, 342–345. https://doi.org/10.12968/denu.2000.27.7.342.
48. Benfante, A.; Ciresi, A.; Bellia, M.; Cannizzaro, F.; Bellia, V.; Giordano, C.; Scichilone, N. Early Lung Function Abnormalities in Acromegaly. *Lung* **2015**, *193*, 393–399. https://doi.org/10.1007/s00408-015-9710-1.
49. Rowe, A.W.; Mortimer, H. Endocrine studies: XLII. A note on acromegaly with the report of a case. *Endocrinology* **1934**, *18*, 20–32. https://doi.org/10.1210/endo-18-1-20.
50. Zafar, A.; Jordan, D.R. Enlarged Extraocular Muscles as the Presenting Feature of Acromegaly. *Ophthalmic Plast. Reconstr. Surg.* **2004**, *20*, 334–336. https://doi.org/10.1097/01.iop.0000132179.74910.79.
51. Duru, N.; Ersoy, R.; Altinkaynak, H.; Duru, Z.; Çağil, N.; Çakir, B. Evaluation of Retinal Nerve Fiber Layer Thickness in Acromegalic Patients Using Spectral-Domain Optical Coherence Tomography. *Semin. Ophthalmol.* **2014**, *31*, 285–290. https://doi.org/10.3109/08820538.2014.962165.
52. Leon-Carrion, J.; Martin-Rodriguez, J.F.; Madrazo-Atutxa, A.; Soto-Moreno, A.; Venegas-Moreno, E.; Torres-Vela, E.; Benito-López, P.; Gálvez, M.Á.; Tinahones, F.J.; Leal-Cerro, A. Evidence of Cognitive and Neurophysiological Impairment in Patients with Untreated Naive Acromegaly. *J. Clin. Endocrinol. Metab.* **2010**, *95*, 4367–4379. https://doi.org/10.1210/jc.2010-0394.
53. Tejera Pérez, C.; Olivier Pascual, N.; Sánchez Bao, A.M.; Arroyo Castillo, M.R. Eye Symptoms in Acromegaly, beyond Visual Field Alteration. Endocrinol. *Diabetes Nutr. Engl. Ed* **2022**, *69*, 309–311. https://doi.org/10.1016/j.endien.2022.03.005.
54. Suzuki, T.; Tokuda, Y. Face the truth: A 76-year-old man with chronic heart failure of unknown origin. *BMJ Case Rep.* **2014**, *2014*, bcr2014204789. https://doi.org/10.1136/bcr-2014-204789.
55. Malicka, J.; Świrska, J.; Nowakowski, A. Familial acromegaly—Case study of two sisters with acromegaly. *Endokrynol. Pol.* **2011**, *62*, 554–557.
56. Tiryakioğlu, Ö.; Caneroğlu, N.Ü.; Yılmaz, E.; Gazioğlu, N.; Kadıoğlu, P.; Açbay, Ö.; Gündoğdu, S. Familial Acromegaly: A Familial Report and Review of the Literature. *Endocr. Res.* **2004**, *30*, 239–245. https://doi.org/10.1081/erc-120039568.

57. Dogansen, S.C.; Tanrikulu, S.; Yalin, G.Y.; Yarman, S. Female gonadal functions and ovarian reserve in patients with acromegaly: Experience from a single tertiary center. *Endocrine* **2018**, *60*, 167–174. https://doi.org/10.1007/s12020-018-1540-5.
58. Inayet, N.; Hayat, J.; Bano, G.; Poullis, A. Gastrointestinal symptoms in acromegaly: A case control study. *World J. Gastrointest. Pharmacol. Ther.* **2020**, *11*, 17–24. https://doi.org/10.4292/wjgpt.v11.i2.17.
59. Colao, A.; Amato, G.; Pedroncelli, A.M.; Baldelli, R.; Grottoli, S.; Gasco, V.; Petretta, M.; Carella, C.; Pagani, G.; Tamburano, G.; et al. Gender- and age-related differences in the endocrine parameters of acromegaly. *J. Endocrinol. Investig.* **2002**, *25*, 532–538. https://doi.org/10.1007/bf03345496.
60. Çapoglu, I.; Yilmaz, A.B.; Ünüvar, N.; Orbak, R.; Aksoy, H.; Yesilyurt, H. Gingival Enlargement in Acromegaly. *Endocrine* **2002**, *18*, 207–210. https://doi.org/10.1385/endo:18:3:207.
61. Saeki, N.; Sunada, S.; Tokunaga, H.; Hoshi, S.; Sunami, K.; Terano, T.; Yamaura, A. Growth hormone secreting adenoma with unusual extension: Coexisting pituitary cyst and its clinical significance. *J. Clin. Neurosci.* **2000**, *7*, 146–147. https://doi.org/10.1054/jocn.1999.0169.
62. Jamjoom, Z.A.B.; Al-Maatouq, M.; Jamjoom, A.-H.B.; Malabarey, T.; Al-Rubeaan, K.; Rahman, N.U.; Baharoon, S. Growth Hormone Secreting Pituitary Adenoma: Clinical Aspects and Surgical Outcome. *Ann. Saudi Med.* **1995**, *15*, 178–182. https://doi.org/10.5144/0256-4947.1995.178.
63. Uchida, K.; Arakawa, Y.; Ohyama, K.; Sirakawa, M.; Tsuji, R.; Yokoyama, M.; Imataka, K.; Sato, M.; Shimizu, Y. Growth Hormone-Secreting Pituitary Adenoma Associated with Primary Moyamoya Disease-Case Report—Case Report. *Neurol. Med.-Chir.* **2003**, *43*, 356–359. https://doi.org/10.2176/nmc.43.356.
64. Sumbul, H.E.; Koc, A.S. Hypertension is Common in Patients with Newly Diagnosed Acromegaly and is Independently Associated with Renal Resistive Index. *High Blood Press. Cardiovasc. Prev.* **2019**, *26*, 69–75. https://doi.org/10.1007/s40292-018-0293-9.
65. Asa, S.L.; Bilbao, J.M.; Kovacs, K.; Linfoot, J.A. Hypothalamic neuronal hamartoma associated with pituitary growth hormone cell adenoma and acromegaly. *Acta Neuropathol.* **1980**, *52*, 231–234. https://doi.org/10.1007/bf00705811.
66. Klijn, J.G.M.; Lamberts, S.W.J.; de Jong, F.H.; van Dongen, K.J.; Birkenhäger, J.C. Interrelationships between tumour size, age, plasma growth hormone and incidence of extrasellar extension in acromegalic patients. *Eur. J. Endocrinol.* **1980**, *95*, 289–297. https://doi.org/10.1530/acta.0.0950289.
67. Foltyn, W.; Kos-Kudla, B.; Strzelczyk, J.; Matyja, V.; Karpe, J.; Rudnik, A.; Marek, B.; Kajdaniuk, D.; Sieron, A.; Latos, W. Is There Any Relation between Hyperinsulinemia, Insulin Resistance and Colorectal Lesions in Patients with Acromegaly? *Neuro Endocrinol. Lett.* **2008**, *29*, 107–112.
68. De Menis, E.; Prezant, T.R. Isolated familial somatotropinomas: Clinical features and analysis of the MEN1 gene. *Pituitary* **2002**, *5*, 11–15. https://doi.org/10.1023/a:1022193015993.
69. Yoshida, N.; Goto, H.; Suzuki, H.; Nagasawa, K.; Takeshita, A.; Okubo, M.; Miyakawa, M.; Mori, Y.; Fukuhara, N.; Nishioka, H.; et al. Ketoacidosis as the initial clinical condition in nine patients with acromegaly: A review of 860 cases at a single institute. *Eur. J. Endocrinol.* **2013**, *169*, 127–132. https://doi.org/10.1530/eje-13-0060.
70. Subramnaian, M.; Shah, V.; Saggu, D.K.; Yalagudri, S.; Narasimhan, C. Looking above the heart: A rare cause of ventricular tachycardia. *J. Arrhythmia* **2021**, *37*, 1120–1122. https://doi.org/10.1002/joa3.12546.
71. Elarabi, A.M.; Mosleh, E.; Alamlih, L.I.; Albakri, M.M.; Ibrahim, W.H. Massive Pulmonary Embolism as the Initial Presentation of Acromegaly: Is Acromegaly a Hypercoagulable Condition? *Am. J. Case Rep.* **2018**, *19*, 1541–1545. https://doi.org/10.12659/ajcr.911572.
72. Lopis, S.; Rubenstein, A.H.; Wright, A.D. Measurements of Serum Growth Hormone and Insulin in Gigantism. *J. Clin. Endocrinol. Metab.* **1968**, *28*, 393–398. https://doi.org/10.1210/jcem-28-3-393.

73. Nagulesparen, M.; Trickey, R.; Davies, M.J.; Jenkins, J.S. Muscle changes in acromegaly. *BMJ* **1976**, *2*, 914–915. https://doi.org/10.1136/bmj.2.6041.914.
74. Ferrer-García, J.C.; Juanes, R.O.; Juan, C.S.; Jiménez, R.; Catalá, J.C.; Ballester, A.H. Miopatía como presentación infrecuente de acromegalia. *Endocrinol. Nutr.* **2007**, *54*, 182–185. https://doi.org/10.1016/s1575-0922(07)71427-4.
75. Lewis, P.D. Neuromuscular Involvement in Pituitary Gigantism. *BMJ* **1972**, *2*, 499–500. https://doi.org/10.1136/bmj.2.5812.499.
76. Arikan, S.; Bahceci, M.; Tuzcu, A.; Gokalp, D. N-terminal pro-brain natriuretic peptide in newly diagnosed acromegaly. *J. Endocrinol. Investig.* **2010**, *33*, 571–575. https://doi.org/10.1007/bf03346650.
77. Howard, G.M.; English, F.P. Occurrence of Glaucoma in Acromegalics. *Arch. Ophthalmol.* **1965**, *73*, 765–768. https://doi.org/10.1001/archopht.1965.00970030767003.
78. Mandel, L.; Zeng, Q.; Silberthau, K.R. Parotid Gland Enlargement in Acromegaly: A Case Report of This Rare Finding. *J. Oral Maxillofac. Surg.* **2020**, *78*, 564–567. https://doi.org/10.1016/j.joms.2019.12.001.
79. Low, P.A.; Mcleod, J.G.; Turtle, J.R.; Donnelly, P.; Wright, R.G. Peripheral neuropathy in acromegaly. *Brain* **1974**, *97*, 139–152. https://doi.org/10.1093/brain/97.1.139.
80. Pokhrel, B.; Khanal, S.; Chapagain, P.; Sedain, G. Pituitary Apoplexy Complicated by Cerebral Infarction: A Case Report. *J. Nepal Med. Assoc.* **2021**, *59*, 723–726. https://doi.org/10.31729/jnma.6120.
81. Roelfsema, F.; Berg, G.V.D.; van Dulken, H.; Veldhuis, J.D.; Pincus, S.M. Pituitary apoplexy in acromegaly, a long-term follow-up study in two patients. *J. Endocrinol. Investig.* **1998**, *21*, 298–303. https://doi.org/10.1007/bf03350332.
82. Mantri, N.M.; Amsterdam, E.; Tan, M.; Singh, G.D. Power Failure: Acromegalic Cardiomyopathy. *Am. J. Med.* **2016**, *129*, 674–677. https://doi.org/10.1016/j.amjmed.2016.02.041.
83. Hashim, Z.; Gupta, M.; Nath, A.; Khan, A.; Neyaz, Z.; Tiwari, S.; Mishra, R.; Srivastava, S.; Gupta, S. Prevalence of sleep apnea and lung function abnormalities in patients with acromegaly. *Lung India* **2022**, *39*, 58–64. https://doi.org/10.4103/lungindia.lungindia_182_21.
84. Heireman, S.; Delaey, C.; Claerhout, I; Decock, C.E. Restrictive extraocular myopathy: A presenting feature of acromegaly. *Indian J. Ophthalmol.* **2011**, *59*, 517. https://doi.org/10.4103/0301-4738.86330.
85. Agrawal, M.; Maitin, N.; Rastogi, K.; Bhushan, R. Seeing the unseen: Diagnosing acromegaly in a dental setup. *BMJ Case Rep.* **2013**, *2013*, bcr2013200266. https://doi.org/10.1136/bcr-2013-200266.
86. Tran, H.A.; Petrovsky, N.; Field, A.J. Severe Diabetic Retinopathy: A Rare Complication of Acromegaly. *Intern. Med. J.* **2002**, *32*, 52–54.
87. Arya, K.R.; Krishna, K.; Chadda, M. Skin manifestations of acromegaly—A study of 34 cases. *Indian J. Dermatol. Venereol. Leprol.* **1997**, *63*, 178–180.
88. Guo, X.; Zhao, Y.; Wang, M.; Gao, L.; Wang, Z.; Zhang, Z.; Xing, B. The posterior pharyngeal wall thickness is associated with OSAHS in patients with acromegaly and correlates with IGF-1 levels. *Endocrine* **2018**, *61*, 526–532. https://doi.org/10.1007/s12020-018-1631-3.
89. Gonzalez, B.; Vargas, G.; Mendoza, V.; Nava, M.; Rojas, M.; Mercado, M. The Prevalence of Colonic Polyps in Patients with Acromegaly: A Case-Control, Nested in a Cohort Colonoscopic Study. *Endocr. Pract.* **2017**, *23*, 594–599. https://doi.org/10.4158/ep161724.or.
90. Imran, S.A.; Aldahmani, K.A.; Penney, L.; Croul, S.E.; Clarke, D.B.; Collier, D.M.; Iacovazzo, D.; Korbonits, M. Unusual AIP mutation and phenocopy in the family of a young patient with acromegalic gigantism. *Endocrinol. Diabetes Metab. Case Rep.* **2018**, *2018*. https://doi.org/10.1530/edm-17-0092.
91. Zangeneh, F.; Carpenter, P.C. Visual Vignette. *Endocr. Pract.* **2002**, *8*, 475. https://doi.org/10.4158/ep.8.6.475.
92. Bolton, K.C.; Gilbert, M.P. Visual Vignette. *Endocr. Pract.* **2018**, *24*, 128. https://doi.org/10.4158/ep-2017-0014.

93. Muthusamy, K.; Bingham, R.J. Visual Vignette. *Endocr. Pract.* **2010**, *16*, 531. https://doi.org/10.4158/ep09336.vv.
